# Lineage Divergence and Vector-Specific Adaptation Have Driven Chikungunya Virus onto Multiple Adaptive Landscapes

**DOI:** 10.1128/mBio.02738-21

**Published:** 2021-11-09

**Authors:** Rubing Chen, Jessica A. Plante, Kenneth S. Plante, Ruimei Yun, Divya Shinde, Jianying Liu, Sherry Haller, Suchetana Mukhopadhyay, Scott C. Weaver

**Affiliations:** a Institute for Human Infections and Immunology, University of Texas Medical Branch, Galveston, Texas, USA; b Center for Tropical Diseases, University of Texas Medical Branch, Galveston, Texas, USA; c Department of Microbiology and Immunology, University of Texas Medical Branch, Galveston, Texas, USA; d World Reference Center for Emerging Viruses and Arboviruses, University of Texas Medical Branch, Galveston, Texas, USA; e Department of Biology, Indiana University, Bloomington, Indiana, USA; CDC

**Keywords:** alphavirus, arbovirus, arthropod vectors, evolution, mosquito

## Abstract

Previous studies have shown that the adaptation of Indian Ocean lineage (IOL) chikungunya virus (CHIKV) strains for Aedes albopictus transmission was mediated by an E1-A226V substitution, followed by either a single substitution in E2 or synergistic substitutions in the E2 and E3 envelope glycoproteins. Here, we examined whether Asian lineage strains, including those that descended from the 2014 Caribbean introduction, are likely to acquire these A. albopictus-adaptive E2 substitutions. Because Asian lineage strains cannot adapt through the E1-A226V substitution due to an epistatic constraint, we first determined that the beneficial effect of these E2 mutations in IOL strains is independent of E1-A226V. We then introduced each of these E2 adaptive mutations into the Asian lineage backbone to determine if they improve infectivity for *A. albopictus*. Surprisingly, our results indicated that in the Asian lineage backbone, these E2 mutations significantly decreased CHIKV fitness in *A. albopictus*. Furthermore, we tested the effects of these mutations in Aedes aegypti and observed different results from those in *A. albopictus*, suggesting that mosquito species-specific factors that interact with the envelope proteins are involved in vector infection efficiency. Overall, our results indicate that the divergence between Asian lineage and IOL CHIKVs has led them onto different adaptive landscapes with differing potentials to expand their vector host range.

## INTRODUCTION

The urbanization of mosquito-borne viral diseases is largely dependent on pathogen adaptation to utilize peridomestic mosquito vectors. Many successful urban viral pathogens, such as dengue virus (DENV), Zika virus (ZIKV), and chikungunya virus (CHIKV), use *Aedes* (*Stegomyia*) *aegypti* and, to some extent, *Aedes* (*Stegomyia*) *albopictus* as their vectors (1, 2). Aedes aegypti is broadly distributed in tropical and subtropical regions throughout the world, whereas A. albopictus can also survive colder climates and thus is found in temperate regions of China, the Americas, Europe, and eastern Australia (3). Additionally, whereas A. aegypti is highly endophilic and prefers taking blood meals from humans, *A. albopictus* is more exophilic and in some locations bites a variety of domestic and wild animals in addition to humans. Vector competence is therefore an important factor dictating the distribution of mosquito-borne viruses, and understanding the mechanism(s) of adaptation to a given vector may allow us to anticipate patterns of spread by mosquito-borne viruses and redirect public health efforts accordingly.

CHIKV is an alphavirus in the family *Togaviridae* and is arguably the most medically important of all alphaviruses due to its widespread distribution, history of explosive outbreaks, and frequency of chronic disease symptoms. CHIKV infection leads to chikungunya fever, accompanied by severe, debilitating, and often chronic arthralgia that has major economic as well as public health impacts (4). Rooted in sub-Saharan African sylvatic transmission cycles, CHIKV strains have emerged at least twice during the past century from the East, Central, South Africa (ECSA) enzootic lineage into Asia, forming independent urban lineages (5). The first lineage identified during the modern scientific era, termed the Asian lineage, was introduced from Africa between 1879 and 1956 and subsequently generated epidemics in Southeast Asia and India. Although not detected in India since 1973, the Asian lineage is still causing outbreaks in Southeast Asia and Oceania. In addition, it was the Asian lineage of CHIKV that first appeared in the Americas via the Caribbean in late 2013 (6), followed by its rapid spread throughout much of Latin America (7–9) as well as Florida (10) and Texas (11) in the United States. The only mosquito species directly incriminated as an urban vector during these American epidemics involving approximately 2.4 million persons has been A. aegypti (12–14), although *A. albopictus* is also present throughout most of the affected region.

The second modern urban CHIKV emergence occurred in 2004 when an ECSA lineage strain initiated an outbreak in coastal Kenya and then independently spread to the Indian Ocean islands and India in 2005 and 2006, respectively (5), forming the epidemic Indian Ocean lineage (IOL). Initial IOL circulation in East Africa relied principally on A. aegypti (15). However, soon after this CHIKV strain reached La Réunion Island, *A. albopictus* began to play a major role in transmission. This vector switch was associated with an A226V substitution in the E1 envelope glycoprotein (16) that was later shown to enhance infection and dissemination in *A. albopictus* (17, 18). Similarly, whereas the initial outbreaks in India were caused by strains with E1-226A (19), E1-226V emerged beginning in 2007 in the state of Kerala, where *A. albopictus* is abundant, and the subsequent distribution of E1-226A versus E1-226V has reflected the abundance of these two vectors (20–22). From India, IOL strains rapidly spread not only through the entire Indian Subcontinent and Southeast Asia but also to Europe and Oceania, where A. aegypti is not abundant (5, 23).

The continued, rapid adaptation of the CHIKV IOL to *A. albopictus* has been attributed to a series of point mutations on all three of the envelope proteins, E1, E2, and E3 (17, 24). Although infection of midgut epithelial cells following an infectious blood meal initiates vector infection (25), it is not clear which stage(s) of the viral replication cycle is directly targeted or altered to determine the expanded vector range mediated by these envelope protein substitutions. The initial adaptation of IOL CHIKV strains to *A. albopictus* occurred via the convergent E1-A226V substitution. This residue is in the E1*ij* loop that is crucial for the pH-sensitive E2/E1 conformational change that exposes the nearby fusion peptide during endosomal entry (26). Although the E1-A226V substitution improves the infectivity of IOL strains for *A. albopictus* by about 40-fold, it has little or no effect on fitness in A. aegypti, suggesting that a mosquito species-specific factor(s) determines the importance of this substitution (17).

Following the spread of different CHIKV IOL sublineages, some of which acquired E1-226V, secondary adaptive mutations in E2 (L210Q and K252Q) or an E2-R198Q/E3-S18F synergistic pair further enhanced infection of *A. albopictus* (24). In addition, an E2-K233E substitution that was not naturally associated with E1-226V also enhances infection of *A. albopictus* in the presence of E1-226V (24). Based on the atomic resolution structure of the CHIKV spike, these E2 mutations are located in or near the acid-sensitive region (ASR) that is critical for triggering E1/E2 disassociation in response to low pH, which initiates E1 trimerization and endosomal membrane fusion (24). Moreover, combining E2-210Q and E2-252Q, or introducing the substitution E2-233E, further enhances infection of *A. albopictus*, indicating that IOL strains can readily explore their complex fitness landscape for additional adaptive pathways to improve infectivity for this widespread mosquito vector. However, as with E1-226V, these E2 mutations do not significantly influence the infectivity of IOL strains in A. aegypti (24), indicating that their beneficial effects in *A. albopictus* are mediated by species-specific factors.

In contrast to the IOL, there is no evidence that the Asian CHIKV lineage has adapted to *A. albopictus* as a primary vector despite circulating in its native territory for at least 60 years. Furthermore, the *A. albopictus*-adaptive E1, E2, and E3 mutations observed in IOL strains have never been detected in an Asian lineage strain. A mutation unique to the Asian lineage, E1-A98T, presumably acquired via a founder effect during its introduction from Africa, imposes an epistatic constraint on the E1-226 residue, thus precluding at least one *A. albopictus*-adaptive pathway of the Asian lineage (27, 48). However, it is unclear why the Asian lineage has not adapted to *A. albopictus* via other mutations such as those in the E2 gene observed in some IOL strains. One possibility is that these E2 mutations are dependent on E1-226V since most of them (except E2-K233E) appeared only after the E1-A226V mutation was fixed in certain IOL sublineages. In addition, an epistatic interaction also occurs between E1-226V and E2-211T, an amino acid located in the same E2 ASR (28), suggesting that epistasis could affect the entire spectrum of E2 vector-adaptive mutations. Alternatively, if the appearance of the *A. albopictus*-adaptive E2 mutations was independent of E1-226V, their later appearance (after E1-A226V) may reflect only their smaller fitness benefit and, hence, lower selection coefficient (∼10-fold versus 40-fold [17, 24]). In that case, these IOL E2 adaptive mutations, namely, E2-198Q, E2-210Q, E2-233E, and E2-252Q, might eventually also be selected by *A. albopictus* in Asian lineage CHIKV strains to enhance transmission efficiency, especially in temperate climates and rural locations.

To test these hypotheses, we engineered CHIKV strains from the IOL and Asian lineage with these specific E2 mutations and evaluated their effects on infection and dissemination in *A. albopictus* and A. aegypti mosquitoes. Our main goals were to (i) determine if the *A. albopictus*-adaptive E2 mutations in IOL strains are dependent on E1-226V, (ii) evaluate the potential of these E2 mutations to emerge in Asian lineage CHIKV as an adaptation to *A. albopictus*, and (iii) determine if these E2 mutations affect infection of A. aegypti.

## RESULTS

### Beneficial effects of IOL E2 *A. albopictus*-adaptive mutations do not depend on E1-226V.

Four *A. albopictus*-adaptive E2 substitutions (E2-R198Q, E2-L210Q, E2-K233E, and E2-K252Q) previously shown to increase fitness in an IOL (SL07) backbone in the context of E1-226V were introduced into the SL07 IOL backbone encoding E1-226A, referred to here as the SL07 wild type (wt). Approximately equal amounts of the wt and E2 mutant viruses as measured by genome ratios, one with an introduced synonymous ApaI/PspOMI restriction site marker and one without, were mixed, and *A. albopictus* mosquitoes were infected via an artificial blood meal. To account for potential bias caused by the marker, reciprocal experiments were conducted where the marker was swapped into the competing backbone. Mosquito heads were collected on day 10, and the differences in wt/mutant ratios in the heads compared to the blood meals were used to assess the mutation’s impact on fitness for infection and dissemination.

**FIG 1:**
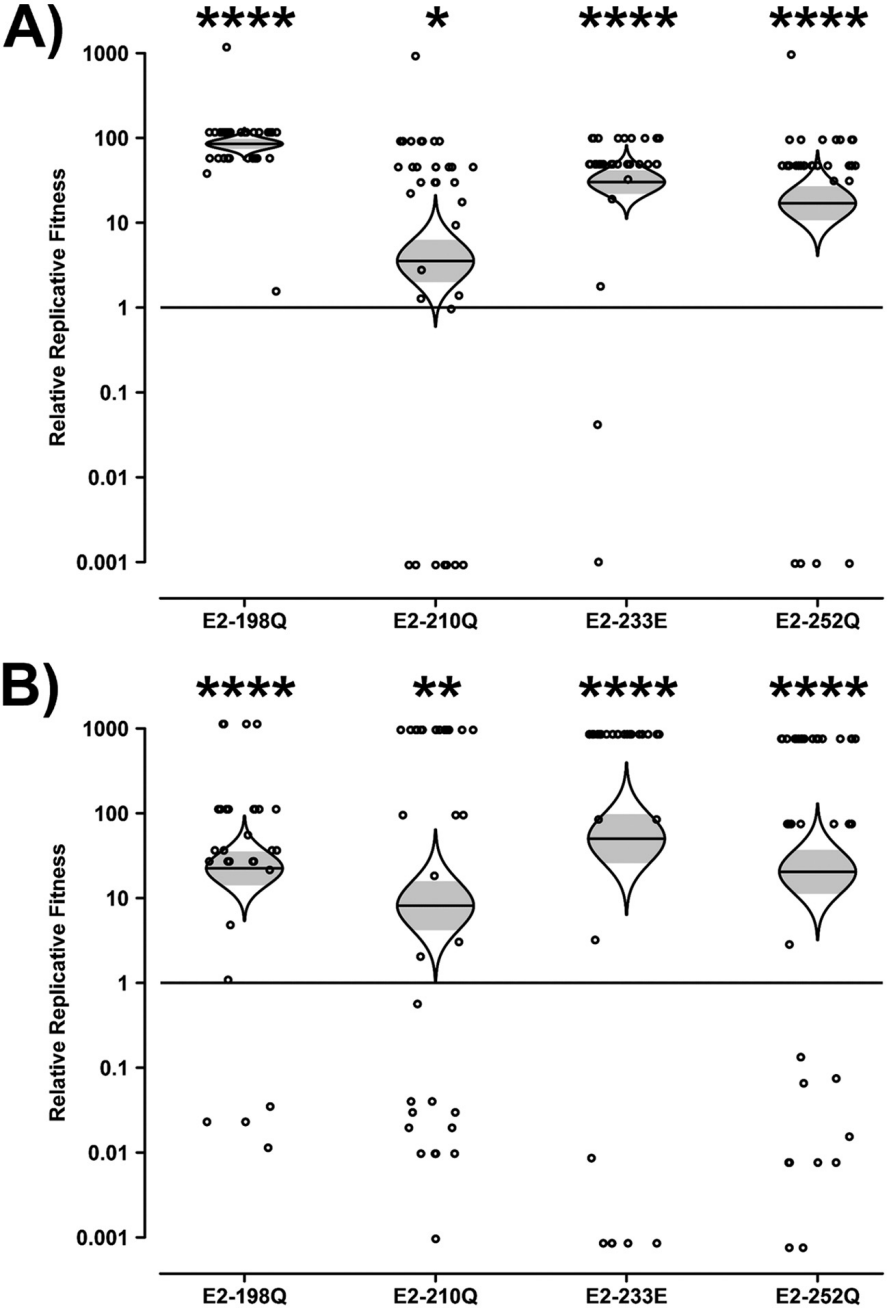
Impact of E2 mutations on the fitness of IOL CHIKV in Aedes albopictus in the absence of E1-226V. Aedes albopictus mosquitoes were fed artificial blood meals with approximately equal genomic ratios of wt and E2 mutant CHIKV SL07, one with an introduced ApaI marker and one without. At 10 dpi, heads were collected, and their virus mixture was passaged for 2 days in Vero cells. The resulting cell culture supernatant was amplified by RT-PCR, and the ratio of ApaI-marked to unmarked CHIKV was determined by restriction digestion. Reciprocal experiments were conducted such that the ApaI marker was present in either the mutant (A) or the wt (B) strain. Relative replicative fitness values were calculated as described in Materials and Methods. The Holm-Sidak correction for multiple *P* values was applied. *, *P* < 0.05; **, *P* < 0.01; ***, *P* < 0.001; ****, *P* < 0.0001.

The majority of mosquito heads had a relatively pure strain composition, with 248 of 265 containing ≥95% of a single competitor strain. This may reflect, in part, the population bottleneck that occurs upon initial midgut infection and the resultant introduction of a stochastic component to the competition outcome. However, the E2 mutations consistently conferred a fitness advantage to SL07 E1-226A in *A. albopictus* (Fig. 1). This advantage was measured regardless of which strain contained the restriction marker, which did not significantly affect the calculated relative replicative fitness values (two-tailed paired *t* test, *t* = 0.4683; df = 3; *P* = 0.6715). The fitness advantage was highly significant for E2-198Q, E2-233E, and E2-252Q (*P* < 0.0001): E2-198Q resulted in an 84- or 22-fold advantage, E2-233E resulted in a 30- or 50-fold advantage, and E2-252Q resulted in a 17- or 20-fold advantage depending on whether the marker was located in the mutant or wt strains, respectively. The E2-210Q mutation, on the other hand, conferred a more modest 4- or 8-fold advantage. These results indicate that the beneficial effects of the E2 *A. albopictus*-adaptive mutations are not contingent upon E1-226V in an IOL backbone.

### IOL E2 *A. albopictus*-adaptive mutations in the Asian lineage are neutral or deleterious in *A. albopictus*.

The E2-198Q, E2-233E, and E2-252Q mutations were introduced into the Asian lineage strain Mal06, which naturally contains E1-226A. Because two point mutations are required to convert L to Q at E2-210 in the Asian lineage, this substitution is considered unlikely in nature and was therefore excluded from the investigation. As was the case in the SL07 backbone, the strain containing the introduced restriction marker did not significantly impact the resulting changes in fitness (two-tailed paired *t* test, *t* = 0.8188; df = 2; *P* = 0.4990), and the vast majority of heads (134 of 146) contained ≥95% of a single strain.

**FIG 2:**
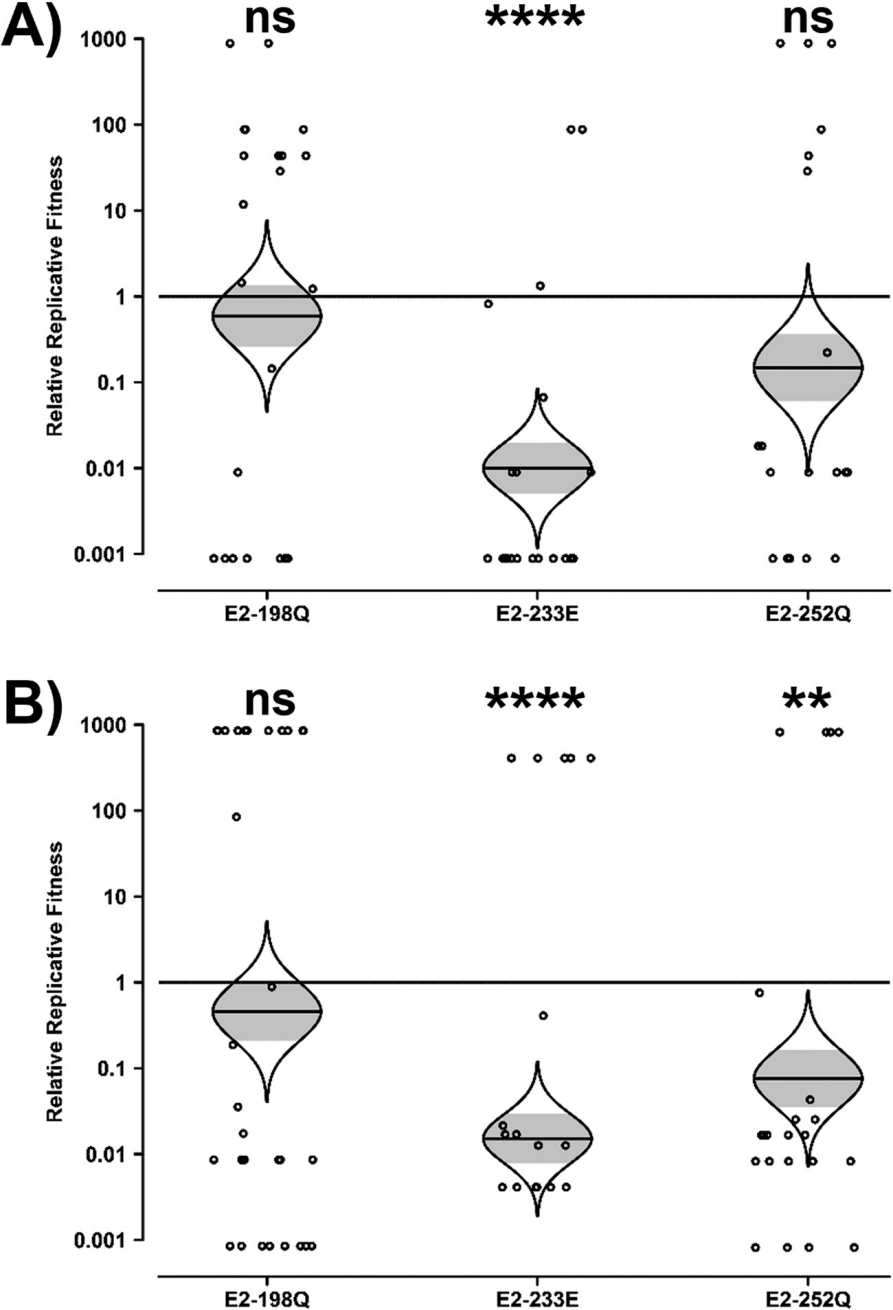
Impact of E2 mutations on the fitness of Asian lineage CHIKV in Aedes albopictus. Aedes albopictus mosquitoes were fed artificial blood meals with approximately equal genomic ratios of wt and E2 mutant CHIKV Mal06, one with an introduced ApaI marker and one without. At 10 dpi, heads were collected, and their virus mixture was passaged for 2 days in Vero cells. The resulting cell culture supernatant was amplified by RT-PCR, and the ratio of ApaI-marked to unmarked CHIKV was determined by restriction digestion. Reciprocal experiments were conducted such that the ApaI marker was present in either the mutant (A) or the wt (B) strain. Relative replicative fitness values were calculated as described in Materials and Methods. The Holm-Sidak correction for multiple *P* values was applied. ns, not significant (*P* ≥ 0.05); **, *P* < 0.01; ****, *P* < 0.0001.

In contrast to the IOL lineage SL07 backbone, the Asian lineage Mal06 backbone was either unaffected or negatively impacted by the E2 mutations (Fig. 2). E2-198Q resulted in no significant fitness change, with a 2-fold decrease in replicative fitness regardless of which strain contained the restriction marker. E2-252Q also resulted in a minimal but negative impact on relative replicative fitness, decreasing fitness by 7-fold or 13-fold depending on whether the marker was in the mutant or the wt strain, respectively. The only mutation to result in a substantial impact in the Mal06 Asian lineage backbone was E2-233E, which decreased fitness by 100- or 50-fold depending on whether the marker was in the mutant or the wt strain, respectively. These data demonstrate that the suite of E2 mutations is not absent from Asian lineages solely because of the inability of the Asian lineage to select E1-226V but also because the E2 mutations themselves are neutral at best and frequently deleterious.

### IOL E2 *A. albopictus*-adaptive mutations in the Asian lineage are neutral or deleterious in A. aegypti.

While the IOL E2 mutations were not advantageous to the Mal06 Asian lineage backbone in *A. albopictus*, their effect in A. aegypti was unknown. Although the IOL E2 mutations conferred no advantage to strain SL07 in A. aegypti (24), the dominance of A. aegypti in Asian lineage CHIKV circulation merits an investigation of their impact in the context of Mal06 in this vector. As described above, a mixture of wt and E2-mutated viruses was fed to mosquitoes in an artificial blood meal. The heads of engorged mosquitoes were collected on day 10, and the differences in wt-to-mutant ratios in the heads compared to the blood meals were measured by Sanger sequencing and used to assess each mutation’s impact on fitness for infection and transmission.

**FIG 3:**
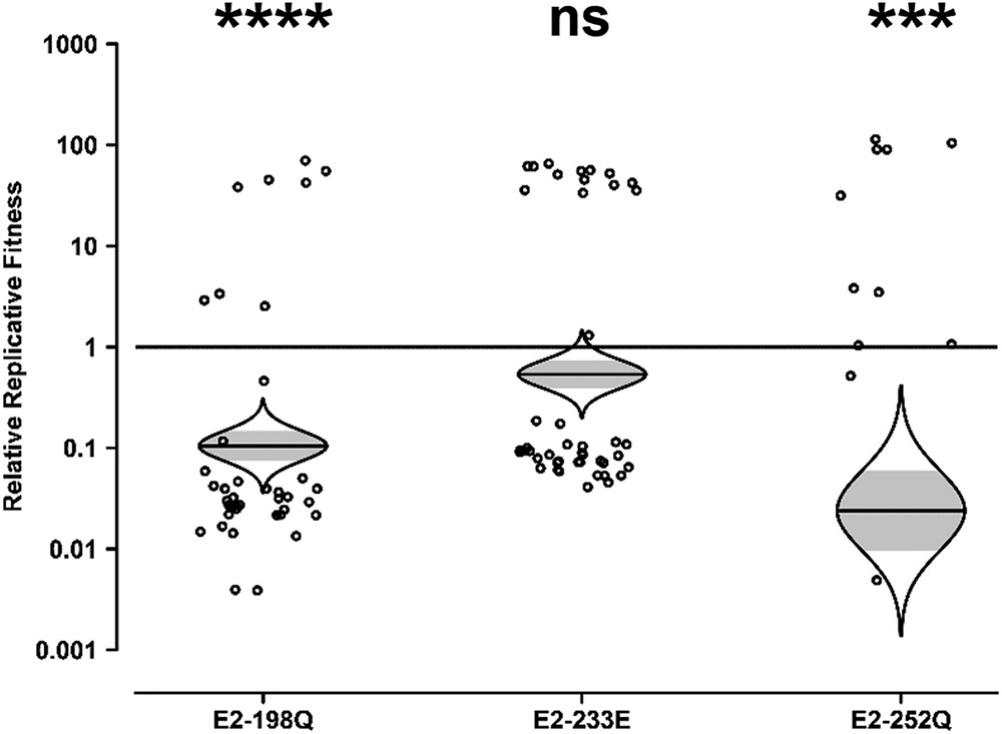
Impact of E2 mutations on the fitness of Asian lineage CHIKV in Aedes aegypti. Aedes aegypti mosquitoes were fed artificial blood meals with approximately equal genomic ratios of wt and E2 mutant CHIKV Mal06. At 10 dpi, heads were collected, and their virus mixture was passaged for 2 days in Vero cells. The resulting cell culture supernatant was amplified by RT-PCR, and the ratio of wt to mutant CHIKV was determined by Sanger sequencing. Relative replicative fitness values were calculated as described in Materials and Methods. The Holm-Sidak correction for multiple *P* values was applied. ns, not significant (*P* ≥ 0.05); ***, *P* < 0.001; ****, *P* < 0.0001.

Consistent with the results in *A. albopictus*, the IOL E2 mutations were either neutral or deleterious in the Mal06 backbone in A. aegypti (Fig. 3). The E2-233E mutation was nearly neutral, resulting in a 2-fold decrease in fitness that did not reach the level of statistical significance. The E2-198Q and E2-252Q mutations, on the other hand, were significantly deleterious, resulting in 9-fold and 50-fold decreases in fitness, respectively. As with the *A. albopictus* experiments assessed by the restriction digest method to estimate strain ratios, the A. aegypti mosquitoes assessed by the Sanger method overwhelmingly (91 of 108) contained ≥95% of a single strain.

### Genetic differences in IOL and Asian lineage CHIKV strains result in contrasting fitness effects of E2 mutations in *A. albopictus*.

**FIG 4:**
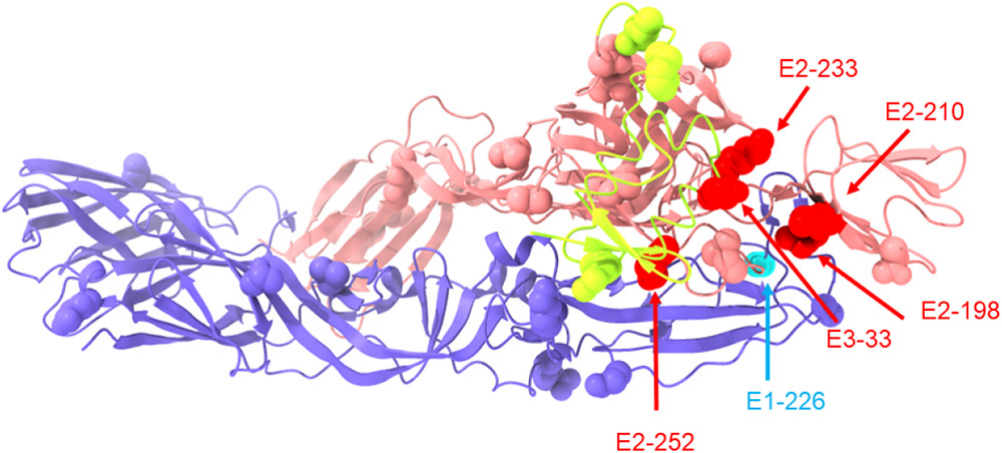
Diversity between IOL and Asian lineage CHIKV envelope glycoproteins. The immature envelope glycoprotein complex of CHIKV (from PDB accession number 3N40) is shown. The E1 protein is in light blue, the E2 protein is in light red, and the E3 protein is in yellow. Residues that differ between SL07 (IOL) and Mal06 (Asian lineage) envelope proteins are shown as spheres. In addition, the IOL adaptive mutations E2-198, E2-210, E2-233, E2-252, as well as E3-33 are shown as spheres and highlighted in red despite their having the same amino acids in the SL07 and Mal06 backbones. Residue E1-226 is shown as spheres highlighted in cyan. Residues of particular interest are highlighted with labels and arrows.

The structures of the glycoproteins (E3, E2, and E1) were examined for potential explanations for the dramatically different fitness effects of the adaptive E2 mutations in IOL versus Asian lineage CHIKV strains. The atomic structure of the IOL glycoproteins, but not the Asian lineage glycoproteins, has been solved (26). Overall, there are 35 amino acids (aa) that differ in the structural proteins between the two lineages, including 4 in E3 (64 aa total), 17 in E2 (423 aa total), 4 in 6K/TF (61 aa total), and 10 in E1 (439 aa total) (Fig. 4; see also Table S1 in the supplemental material). *In silico*, we mutated residues from the IOL strain to those in the Asian lineage strain. Based on the predicted structures, all amino acids in E2 that differ between the two strains lie along the top of the E2 protein in domains A and B, suggesting that they likely affect host cell binding. Some of the E1 amino acids that differ between these strains are clustered in the hinge region between domains I and II, which is predicted to move during the fusion process (29, 30).

10.1128/mBio.02738-21.5 TABLE S1 Distance in angstroms between amino acid residues that differ between Mal06 and SL07 glycoproteins. Download Table S1, PDF file, 0.07 MB. Copyright © 2021 Chen et al. 2021 Chen et al. https://creativecommons.org/licenses/by/4.0/ This content is distributed under the terms of the Creative Commons Attribution 4.0 International license.

We specifically investigated amino acids in the two strains that might interact with adaptive residues in E2 positions 198, 233, and 252. All residues within 4 Å of these E2 positions, and therefore most likely to be involved in direct interactions, were conserved between the two CHIKV strains. When the potential interaction radius was expanded to 20 Å to identify residues that might be interacting during a conformational change during the assembly or fusion process, 8 residues spanning all three envelope glycoproteins were identified (Table S2).

10.1128/mBio.02738-21.6 TABLE S2 Envelope protein amino acids that differ between IOL (SL07) and Asian lineage (Mal06) CHIKV strains and potentially interact with the IOL adaptive E2 mutations. Download Table S2, PDF file, 0.03 MB. Copyright © 2021 Chen et al. 2021 Chen et al. https://creativecommons.org/licenses/by/4.0/ This content is distributed under the terms of the Creative Commons Attribution 4.0 International license.

Only one residue, E3-33, which exists as E3-33E in the IOL (SL07) strain and as E3-33K in the Asian lineage (Mal06) strain, was identified as a site of potential long-range interactions with all four E2 adaptive mutations. In the native IOL strain, E3-33E and E2-233K are 10 Å apart and consist of oppositely charged amino acids, representing a likely attractive interaction. In the presence of the IOL adaptive substitution pair (E3-33E/E2-233E), the presence of two like charges may weaken this interaction in a way that promotes infectivity or assembly. In Asian lineage strains, the wt E3-33K/E2-233K residues may be repelled based on like charges, in a manner similar to that of the adaptive E3-33E/E2-233E IOL strain; in the presence of the introduced adaptive E2 mutation, the Asian pair becomes E3-33K/E2-233E, a potentially attractive interaction similar to the wt IOL strain. Thus, in both the IOL and Asian lineages, repelling charges between E3-33 and E2-233 appear to confer a fitness advantage over attractive charges.

**FIG 5:**
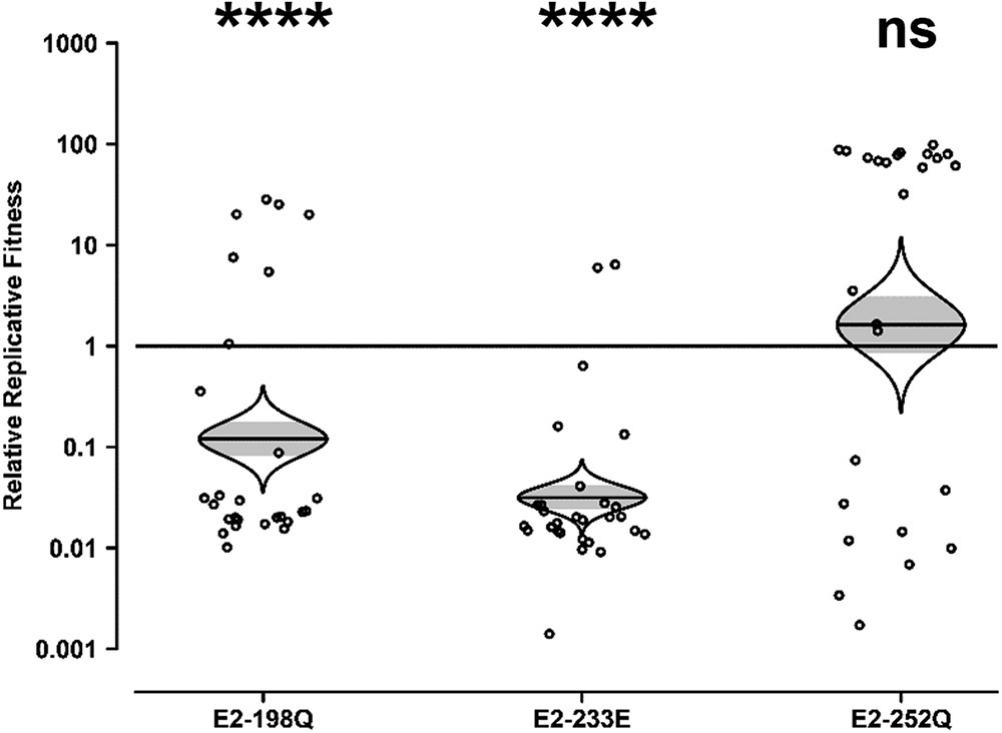
Impact of E2 mutations on the fitness of Asian lineage CHIKV in Aedes albopictus in the context of E3-33E. Aedes albopictus mosquitoes were fed artificial blood meals with approximately equal genomic ratios of wt and E2 mutant CHIKV Mal06, both containing an introduced E3-33E mutation. At 10 dpi, heads were collected, and their virus mixture was passaged for 2 days in Vero cells. The resulting cell culture supernatant was amplified by RT-PCR, and the ratio of wt to mutant CHIKV was determined by Sanger sequencing. Relative replicative fitness values were calculated as described in Materials and Methods. The Holm-Sidak correction for multiple *P* values was applied. ns, not significant (*P* ≥ 0.05); ****, *P* < 0.0001.

To test this hypothesis, E3-33E was introduced into the Asian Mal06 strains with and without each E2 mutation, and their fitness effects in *A. albopictus* were compared using the competition assay with Sanger sequencing (Fig. 5). The E2-198Q mutation, which was neutral in the context of its native E3-33K backbone, was deleterious in the context of E3-33E and resulted in an 8-fold decrease in replicative fitness. The E2-233E mutation, which was highly deleterious in its native E3-33K backbone, was also highly disadvantaged in the context of E3-33E, resulting in a 33-fold decrease in replicative fitness. Finally, E2-252Q, which caused a modest decrease in fitness in its native E3-33K backbone, became neutral in the E3-33E backbone, causing only a 2-fold increase in fitness that failed to attain statistical significance. As with the other competition assays, most mosquito heads (69 of 83) contained ≥95% of a single strain.

## DISCUSSION

CHIKV has expanded its distribution in recent years through the emergence of the IOL from the ECSA lineage from 2005 to 2006, the expansion of the Asian lineage into the Americas in 2013, and the introduction of an ECSA strain from Angola into Brazil in 2014 (31). Whereas the evolution of the IOL has been characterized in part by sequential adaptation to a widespread and invasive mosquito vector, *A. albopictus*, there is no evidence that the Asian lineage has undergone comparable adaptation to any mosquito vector. Given the largely sympatric distributions of A. aegypti and *A. albopictus* (32) as well as the increasing cocirculation of different CHIKV lineages (33), understanding the vector usage and the potential for adaptation to expand vector host range, as well as their underlying mechanisms and constraints, is essential for the prevention and control of future epidemics.

We sought to understand why the Asian CHIKV lineage has not expanded its urban vector usage to *A. albopictus*. Specifically, can the Asian lineage CHIKV strains adopt the same evolutionary pathways as the IOL strains to adapt for *A. albopictus* transmission? To address this question in a feasible manner, capitalizing on previous work, we initially focused on envelope glycoprotein substitutions previously shown in IOL strains to enhance CHIKV infection of *A. albopictus*.

First, our study showed that the secondary adaptive E2 mutations observed in different IOL sublineages do not require the presence of the primary E1-A226V substitution for their fitness advantages in *A. albopictus* in an IOL backbone. Therefore, the adaptation of IOL strains to *A. albopictus* may be mediated by multiple mechanisms during viral infection and replication. *In vitro* experiments have shown that the E1-A226V substitution leads to an increased requirement for membrane cholesterol and a lower endocytic pH for fusion in *A. albopictus* C6/36 cells (34), although it is unclear whether and how these two effects differ between *A. albopictus* and A. aegypti. The IOL *A. albopictus*-adaptive E2 substitutions, located in domain B (residues 198 and 210) and the ASR (residues 233 and 252), likely affect the pH-dependent conformational change that exposes the fusion loop on E1. Although the two sets (domain B and ASR) of mutations are both hypothesized to affect the fusion and cell entry of IOL CHIKV strains in *A. albopictus*, they may have different mechanisms.

Second, our results demonstrate that the IOL and Asian CHIKV lineages have diverged onto different adaptive landscapes for *A. albopictus*. Unlike the IOL lineage, when we introduced the E2 adaptive mutations (E2-R198Q, E2-K233E, and E2-K252Q) into the Asian lineage strain Mal06, fitness was either unchanged or reduced in *A. albopictus*. The functions of E2-K233E and E2-K252Q, both located in the E2 ASR, could include interactions with cellular factors specific to *A. albopictus*, which may regulate the structural change of the ASR under low endosomal pH. In IOL strains, these interactions are advantageous, but in Asian strains, these interactions may be abolished or constrained.

These adaptive E2 mutations in the IOL strains were previously shown to have little or no effect on viral fitness in A. aegypti (24). Our data revealed that these mutations are either neutral or detrimental in the context of Asian lineage CHIKV infection of A. aegypti. Specifically, in the Mal06 backbone, E2-198Q was neutral in *A. albopictus* and moderately deleterious in A. aegypti, E2-233E was strongly deleterious in *A. albopictus* and neutral in A. aegypti, and E2-252Q was moderately deleterious in *A. albopictus* and strongly deleterious in A. aegypti. These results confirm that residues E2-198, −233, and −252 interact with *Aedes* mosquitoes in a species-specific manner. Research to date on CHIKV entry into mosquito cells is largely based on ECSA/IOL strains in cultured *A. albopictus* C6/36 cells (34–37). Comparative studies between the multiple lineages and in both *A. albopictus* and A. aegypti cell lines could enhance the understanding of CHIKV-vector interactions.

Our studies of the E3-33 substitution suggest that, despite its position as the only residue to both differ between SL07 and Mal06 and to be located within 20 Å of all four E2 adaptive residues, it is not responsible for the contrasting fitness effects of these E2 mutations on IOL or Asian lineage CHIKV. Although E3-33 was considered the most likely candidate based on proximity to all E2 adaptive mutations in the mature glycoprotein spike, it is possible that the change(s) responsible for the divergent SL07 and Mal06 phenotypes is more pertinent to viral assembly or fusion and thus is not reflected in the conformation of a mature virion. It is also possible that multiple mutations jointly shape the conformation of the protein(s) during different stages of replication or influence its interaction with cellular factors in *A. albopictus*. None of the E2 residues that we studied contact the Mxra8 receptor identified for several alphaviruses (38). However, this receptor occurs only in vertebrates, so CHIKV interactions with another receptor could be involved in mosquito infection. Regardless, further work is required to fully define the differentiating factors that confine Asian lineage and IOL CHIKV to different adaptive landscapes.

There are several limitations of our studies. We focused only on mutations identified previously in IOL strains as they spread through Asia following the 2005 emergence. However, it is possible that other genome regions have also contributed to CHIKV fitness for transmission by *A. albopictus*, so further studies starting with chimeric viruses mixing other genes among early- and late-stage IOL strains as well as Asian strains may yield further insights.

In conclusion, our results suggest that the IOL and Asian lineage CHIKVs have diverged onto different evolutionary trajectories and fitness landscapes and no longer share the same adaptability for the two urban vectors. Given that CHIKV had no known exposure to *A. albopictus* in Africa prior to 2006 (39), it is unclear whether its ability to readily adapt to this mosquito is a consequence of its ancestral state. It is also unclear why the Asian lineage has lost or never gained the ability to use *A. albopictus* as a major vector in Southeast Asia, where this mosquito is native and abundant. While we cannot exclude the possibility that Asian lineage CHIKV strains could adapt to *A. albopictus* through other mechanisms yet to be realized, our results strongly indicate that any such adaptation must arise through a different mutation or series of mutations than those found in IOL strains.

## MATERIALS AND METHODS

### Cell culture.

Vero African green monkey kidney cells (ATCC CCL-81; American Type Culture Collection, Manassas, VA) were maintained in Dulbecco’s modified Eagle’s essential medium (DMEM) supplemented with 5% fetal bovine serum (FBS) and penicillin/streptomycin at 37°C under 5% CO_2_. C7/10 *A. albopictus* cells were maintained in DMEM supplemented with 10% FBS, 5% tryptose phosphate broth, and penicillin/streptomycin at 28°C under 5% CO_2_.

### Construction of infectious cDNA clones.

Plasmids encoding two wild-type CHIKV strains, SL07 and Mal06, from the IOL and Asian lineage, respectively, were described previously (27). Both wt plasmids contain their ancestral E1-226A codons. Single mutations representing all four of the E2 substitutions (E2-R198Q, E2-L210Q, E2-K233E, and E2-K252Q) were introduced into the SL07 plasmid, and three of the E2 substitutions (E2-R198Q, E2-K233E, and E2-K252Q) in either the presence or absence of an additional E3-K33E substitution were introduced into the Mal06 plasmid using conventional PCR-based methods (40). In addition, to compare relative fitness levels using a restriction digest-based competition test, a synonymous point mutation was introduced into the nsP4 gene in each plasmid, including all of the wt strains and mutants, to form restriction sites cleavable by the endonucleases ApaI and PspOMI (27). All PCR-generated genome regions used for cloning were Sanger sequenced to verify their integrity. Plasmids were purified by centrifugation in cesium chloride gradients.

### Rescue of viruses from infectious clones.

To generate infectious RNA, plasmids were linearized with the NotI restriction endonuclease, followed by *in vitro* transcription from the SP6 promoter as described previously (40). Approximately 10 μg of RNA was electroporated into C7/10 cells. Cell culture supernatants were harvested at 24 to 48 h postelectroporation and stored at −80°C. Infectious viral titers were determined by plaque assays on Vero cells (titers ranged from 10^7^ to 10^8^ PFU/ml). Viruses recovered from electroporated cells were used directly without additional passages.

### Mosquito feeds and harvests.

To determine the relative fitness levels of mutant versus wt CHIKV strains, competition tests were conducted in mosquitoes as described previously (27). To achieve the approximately 1:1 ratio desired for the competition assay, wt and mutant viruses were initially combined at a 1:1 ratio as determined by Vero cell PFU. This ratio, as well as a number of ratios closely flanking it, was further refined to achieve a 1:1 genome copy ratio because the final output assays, whether restriction digest based or PCR based, are all genome-based assays. In the case of experiments with restriction digests as their final output, this adjustment was based on the brightness of reverse transcription-PCR (RT-PCR) amplicon bands. In the case of experiments with Sanger sequencing as their final output, the ratio of the two viruses was estimated based on the single nucleotide polymorphism (SNP) heights in the electropherogram generated by Sanger sequencing. Initial ratios measured genetically varied from 1:1.00 to 1:3.04 (mean = 1:1.40; median = 1:1.17).

The competing viruses were mixed at the determined 1:1 genomic ratio with defibrinated sheep blood to a final concentration of 1 × 10^6^ to 1 × 10^7^ PFU/ml. The blood meal was offered for approximately 1 h to A. aegypti or *A. albopictus* mosquitoes from colonies established from mosquito eggs collected in Bangkok, Thailand. This colony was chosen to correspond to a location where selection for adaptation to both mosquito vectors has presumably occurred for decades. Fully engorged mosquitoes were incubated at 28°C (a typical mean tropical temperature in locations where CHIKV is endemic) with 10% sucrose and 80% relative humidity under a 16-h-light/8-h-dark photoperiod.

Mosquitoes were harvested at 10 days postinfection (dpi). The heads were separated from the bodies and homogenized in 500 μl of DMEM. Heads were selected rather than salivary glands to permit the efficient harvest of large numbers of samples. As salivary gland infection and escape barriers have not been described for CHIKV and none of the mutants studied appear to affect salivary gland replication (41), the CHIKV mixture present in the head is assumed to accurately reflect the CHIKV mixture present for transmission in the saliva.

To determine if these mosquito heads were infected, homogenized samples were passaged in Vero cells prepared in 96-well plates. Supernatants from Vero cells showing cytopathic effect (CPE) were collected at 2 dpi and used for determining the ratio of the competing viruses. Analysis of the Vero cell-passaged ratios was undertaken to screen for infected heads more efficiently than using the more complex and less sensitive RT-PCR. Also, amplification on Vero cells minimized the risk of direct RT-PCR amplicons from saliva being near the limit of detection and thus representing a very small number of CHIKV genomes sampled, compared to the greater sensitivity of Vero cells to amplify more viral genomes. Passaging known mixtures of CHIKV wt and mutant strains in Vero cells in duplicate produced minimal differences in input versus output ratios, and none of the differences were statistically significant whether considering individual strain combinations (*P* value range of 0.617811 to 0.904460 by repeated-measures one-way analysis of variance [ANOVA] comparing the input and duplicate passage outputs at three ratios per strain with a Holm-Sidak correction for multiple comparisons) or the combined data set (*P* = 0.300570) (see Fig. S1 in the supplemental material).

10.1128/mBio.02738-21.1 FIG S1 Passage of competing viruses in Vero cells. All competing groups were mixed in 3 different ratios and passaged in Vero cells in 96-well plates in duplicate for 48 h. RNAs from the initial mix and passaged material were purified, amplified via RT-PCR, and restriction digested as described in Materials and Methods. Gel lanes are labeled I for the input and 1 and 2 for the duplicate passages. Proportions of DNA as estimated by GelQuant.NET software are shown in red next to the relevant bands. Download FIG S1, PDF file, 0.5 MB. Copyright © 2021 Chen et al. 2021 Chen et al. https://creativecommons.org/licenses/by/4.0/ This content is distributed under the terms of the Creative Commons Attribution 4.0 International license.

### Determination of competition outcomes.

Viral RNA was purified from each passaged mosquito head as well as from the initial blood meal using the QIAamp viral RNA kit (Qiagen). Ratios of wild-type and mutant viruses were determined by one of two methods: restriction digestion or Sanger sequencing of RT-PCR amplicons.

For samples evaluated by restriction digestion, the genome region from nucleotides (nt) 6106 to 6794, which includes in its center the introduced ApaI/PspOMI restriction site marker, was amplified by RT-PCR using the Qiagen OneStep RT-PCR kit with the primers 41855-nsF5 (5′-ATATCTAGACATGGTGGA-3′) and 41855-nsR1 (5′-TATCAAAGGAGGCTATGTC-3′). The amplicons were digested with the restriction endonucleases ApaI and PspOMI (which are isoschizomers sharing the recognition site GGGCCC) for 30 min at 27°C followed by 4 h at 37°C. Specificity and complete digestion were confirmed using controls that contained only one of the competing viruses (with or without the marker). Band ratios were determined using GelQuant.NET software (Biochem Lab Solutions). Gel images, along with the corresponding band ratios, are available in Fig. S2. Validation of the restriction digest method is available in Fig. S3.

10.1128/mBio.02738-21.2 FIG S2 Gel images of competition test results. The raw data corresponding to Fig. 1 and 2 are shown here. For each gel picture, the digestion result of the initial mix of viruses is shown in the first lane (I). The following lanes show the results in each mosquito sample (M#). Proportions of DNA as estimated by GelQuant.NET software are shown in red next to the relevant bands. Download FIG S2, PDF file, 0.7 MB. Copyright © 2021 Chen et al. 2021 Chen et al. https://creativecommons.org/licenses/by/4.0/ This content is distributed under the terms of the Creative Commons Attribution 4.0 International license.

10.1128/mBio.02738-21.3 FIG S3 Validation of restriction digest-based genome ratio assessments. CHIKV SL07 RNA, either the wild type containing no restriction enzyme marker or with the E2-R198Q mutation and the ApaI marker, was extracted from viral stocks and combined at four different ratios. The RNA mixtures were subjected to RT-PCR and restriction digest analysis as described in Materials and Methods. Estimated ratios (on the basis of the RNA input as determined by empirically determining the ratio required for a 50:50 observed banding pattern and then scaling the ratios to 5:95, 10:90, 95:5, and 90:10) and observed ratios (on the basis of GelQuant.NET software) are noted above and below the relevant bands. Unpaired two-tailed Student’s *t* test of the mutant/wild-type band ratios found no statistically significant difference between the estimated values and the observed values (*P* = 0.530101 [not significant {ns}]). Download FIG S3, PDF file, 0.09 MB. Copyright © 2021 Chen et al. 2021 Chen et al. https://creativecommons.org/licenses/by/4.0/ This content is distributed under the terms of the Creative Commons Attribution 4.0 International license.

Because control experiments demonstrated that the restriction marker mutation described above unexpectedly affected fitness in A. aegypti (it had been shown to have no phenotype in *A. albopictus*), we switched to using a Sanger sequencing method to estimate mutant/wild-type ratios. This method avoids potential effects on the fitness of the engineered restriction site marker and has been shown in other studies to be highly accurate and reproducible (42–44, 49). The regions containing the mutations of interest were amplified by RT-PCR using the primers Ch18(+) 8730 (5′-TTGGACYAAGCTGCG-3′) and Ch19(−) 9419 (5′-GTCGGATGGTCAGGATACAG-3′). The amplicons were subsequently purified by a Qiagen PCR purification kit and sequenced using a BigDye Terminator v3.1 cycle sequencing kit and an ABI Prism model 3100 genetic analyzer (Applied Biosystems, Foster City, CA). The ratio of the two viruses was estimated based on the SNP heights in the sequencing electropherogram using QSVanalyser software (45). This method has been shown to be highly accurate and reproducible for RNA viruses (42–44). Validation of the Sanger method is available in Fig. S4.

10.1128/mBio.02738-21.4 FIG S4 Validation of Sanger-based genome ratio assessments. Aedes albopictus (Thailand) mosquitoes were fed chikungunya virus (CHIKV) mixtures of wild-type strain SL07 and either the E2-R198Q, E2-L210Q, E2-K333E, or E2-K252Q mutant at approximately equal ratios (mean = 1:1.31; median = 1:1.17) and a combined concentration of 1 × 10^7^ PFU/ml. After 10 days, bodies and heads were harvested. Bodies were screened for the presence of CHIKV by a CPE assay. Eight heads per cohort were selected, each with a CHIKV-positive body. RNA was extracted from the heads and subjected to both Sanger sequencing (as described in Materials and Methods) and pyrosequencing. The correlations between each method’s detected percentages of wild-type and mutant genomes were assessed by linear regression and Spearman’s correlation. Download FIG S4, PDF file, 0.1 MB. Copyright © 2021 Chen et al. 2021 Chen et al. https://creativecommons.org/licenses/by/4.0/ This content is distributed under the terms of the Creative Commons Attribution 4.0 International license.

Using these assays, the frequencies of minority genotypes below an expected frequency of 10% may be observed with variation as high as 20%. However, the variability of accurately measuring low-frequency genotypes does not impact our determination of which genotype is dominant in a sample.

### Molecular modeling.

The structure of the immature envelope glycoprotein complex of a CHIKV IOL strain (PDB accession number 3N40) was used in PyMOL (PyMOL molecular graphics system version 1.8; Schrödinger, LLC, New York, NY) to determine amino acid distances. Figures were generated in UCSF ChimeraX version 1.2.5 (46, 47).

### Statistical analysis.

Relative replicative fitness was calculated as previously described (42, 43). Briefly, replicative fitness values were modeled as *w* = *f*_0_/*i*_0_, where *i*_0_ is the initial ratio of a given strain in the blood meal and *f*_0_ is the final ratio of that strain in the passaged mosquito head. The model was fitted to log_10_(mosquito head output/blood meal input) ∼ strain, with log_10_ conversion performed to improve the normality of the data set. Relative replicative fitness values were determined by the model’s strain coefficient, which was transformed to the original scale as 10^coefficient^. When the band ratios following restriction digestion were 100 to 0, the ratios were considered 99.9 to 0.1 for the calculation of relative replicative fitness to avoid dividing by 0. Similarly, if one of the peak heights following Sanger sequencing was 0, it was changed to 0.1 to avoid dividing by 0 (in this instance, the peak height of the other strain was not changed). The Holm-Sidak method was used to correct for multiple comparisons when considering the *P* values generated by the relative replicative fitness calculations.

## References

[1] Weaver SC, Reisen WK. 2010. Present and future arboviral threats. Antiviral Res 85:328–345. doi:10.1016/j.antiviral.2009.10.008. 19857523 PMC2815176

[2] Petersen LR, Jamieson DJ, Powers AM, Honein MA. 2016. Zika virus. N Engl J Med 374:1552–1563. doi:10.1056/NEJMra1602113. 27028561

[3] Brady OJ, Golding N, Pigott DM, Kraemer MU, Messina JP, Reiner RC, Jr, Scott TW, Smith DL, Gething PW, Hay SI. 2014. Global temperature constraints on Aedes aegypti and Ae. albopictus persistence and competence for dengue virus transmission. Parasit Vectors 7:338. doi:10.1186/1756-3305-7-338. 25052008 PMC4148136

[4] Weaver SC, Lecuit M. 2015. Chikungunya virus and the global spread of a mosquito-borne disease. N Engl J Med 372:1231–1239. doi:10.1056/NEJMra1406035. 25806915

[5] Volk SM, Chen R, Tsetsarkin KA, Adams AP, Garcia TI, Sall AA, Nasar F, Schuh AJ, Holmes EC, Higgs S, Maharaj PD, Brault AC, Weaver SC. 2010. Genome-scale phylogenetic analyses of chikungunya virus reveal independent emergences of recent epidemics and various evolutionary rates. J Virol 84:6497–6504. doi:10.1128/JVI.01603-09. 20410280 PMC2903258

[6] Leparc-Goffart I, Nougairede A, Cassadou S, Prat C, de Lamballerie X. 2014. Chikungunya in the Americas. Lancet 383:514. doi:10.1016/S0140-6736(14)60185-9. 24506907

[7] Nunes MRT, Faria NR, de Vasconcelos JM, Golding N, Kraemer MUG, de Oliveira LF, Azevedo RDSDS, da Silva DEA, da Silva EVP, da Silva SP, Carvalho VL, Coelho GE, Cruz ACR, Rodrigues SG, Vianez JLDSG, Jr, Nunes BTD, Cardoso JF, Tesh RB, Hay SI, Pybus OG, Vasconcelos PFDC. 2015. Emergence and potential for spread of chikungunya virus in Brazil. BMC Med 13:102. doi:10.1186/s12916-015-0348-x. 25976325 PMC4433093

[8] Wang C, Saborio S, Gresh L, Eswarappa M, Wu D, Fire A, Parameswaran P, Balmaseda A, Harris E. 2016. Chikungunya virus sequences across the first epidemic in Nicaragua, 2014-2015. Am J Trop Med Hyg 94:400–403. doi:10.4269/ajtmh.15-0497. 26643533 PMC4751928

[9] Kautz TF, Diaz-Gonzalez EE, Erasmus JH, Malo-Garcia IR, Langsjoen RM, Patterson EI, Auguste DI, Forrester NL, Sanchez-Casas RM, Hernandez-Avila M, Alpuche-Aranda CM, Weaver SC, Fernandez-Salas I. 2015. Chikungunya virus as cause of febrile illness outbreak, Chiapas, Mexico, 2014. Emerg Infect Dis 21:2070–2073. doi:10.3201/eid2111.150546. 26488312 PMC4622247

[10] Kuehn BM. 2014. Chikungunya virus transmission found in the United States: US health authorities brace for wider spread. JAMA 312:776–777. doi:10.1001/jama.2014.9916. 25120064

[11] Texas Department of State Health Services 2016. DSHS announces first Texas-acquired chikungunya case. Texas Department of State Health Services, Austin, TX. https://www.dshs.texas.gov/news/releases/2016/20160531.aspx. Accessed 11 July 2021.

[12] Morrison TE. 2014. Reemergence of chikungunya virus. J Virol 88:11644–11647. doi:10.1128/JVI.01432-14. 25078691 PMC4178719

[13] Diaz-Gonzalez EE, Kautz TF, Dorantes-Delgado A, Malo-Garcia IR, Laguna-Aguilar M, Langsjoen RM, Chen R, Auguste DI, Sanchez-Casas RM, Danis-Lozano R, Weaver SC, Fernandez-Salas I. 2015. First report of Aedes aegypti transmission of chikungunya virus in the Americas. Am J Trop Med Hyg 93:1325–1329. doi:10.4269/ajtmh.15-0450. 26416113 PMC4674253

[14] Dzul-Manzanilla F, Martinez NE, Cruz-Nolasco M, Gutierrez-Castro C, Lopez-Damian L, Ibarra-Lopez J, Martini A, Torres-Leyva J, Bibiano-Marin W, Tornez-Benitez C, Ayora-Talavera G, Manrique-Saide P. 2015. Arbovirus surveillance and first report of chikungunya virus in wild populations of Aedes aegypti from Guerrero, Mexico. J Am Mosq Control Assoc 31:275–277. doi:10.2987/moco-31-03-275-277.1. 26375910

[15] Chretien J-P, Anyamba A, Bedno SA, Breiman RF, Sang R, Sergon K, Powers AM, Onyango CO, Small J, Tucker CJ, Linthicum KJ. 2007. Drought-associated chikungunya emergence along coastal East Africa. Am J Trop Med Hyg 76:405–407. doi:10.4269/ajtmh.2007.76.405. 17360859

[16] Schuffenecker I, Iteman I, Michault A, Murri S, Frangeul L, Vaney MC, Lavenir R, Pardigon N, Reynes JM, Pettinelli F, Biscornet L, Diancourt L, Michel S, Duquerroy S, Guigon G, Frenkiel MP, Brehin AC, Cubito N, Despres P, Kunst F, Rey FA, Zeller H, Brisse S. 2006. Genome microevolution of chikungunya viruses causing the Indian Ocean outbreak. PLoS Med 3:e263. doi:10.1371/journal.pmed.0030263. 16700631 PMC1463904

[17] Tsetsarkin KA, Vanlandingham DL, McGee CE, Higgs S. 2007. A single mutation in chikungunya virus affects vector specificity and epidemic potential. PLoS Pathog 3:e201. doi:10.1371/journal.ppat.0030201. 18069894 PMC2134949

[18] Vazeille M, Moutailler S, Coudrier D, Rousseaux C, Khun H, Huerre M, Thiria J, Dehecq JS, Fontenille D, Schuffenecker I, Despres P, Failloux AB. 2007. Two chikungunya isolates from the outbreak of La Reunion (Indian Ocean) exhibit different patterns of infection in the mosquito, Aedes albopictus. PLoS One 2:e1168. doi:10.1371/journal.pone.0001168. 18000540 PMC2064959

[19] Arankalle VA, Shrivastava S, Cherian S, Gunjikar RS, Walimbe AM, Jadhav SM, Sudeep AB, Mishra AC. 2007. Genetic divergence of chikungunya viruses in India (1963-2006) with special reference to the 2005-2006 explosive epidemic. J Gen Virol 88:1967–1976. doi:10.1099/vir.0.82714-0. 17554030

[20] Sreekumar E, Issac A, Nair S, Hariharan R, Janki MB, Arathy DS, Regu R, Mathew T, Anoop M, Niyas KP, Pillai MR. 2010. Genetic characterization of 2006-2008 isolates of chikungunya virus from Kerala, South India, by whole genome sequence analysis. Virus Genes 40:14–27. doi:10.1007/s11262-009-0411-9. 19851853 PMC7088544

[21] Santhosh SR, Dash PK, Parida M, Khan M, Rao PV. 2009. Appearance of E1: A226V mutant chikungunya virus in coastal Karnataka, India during 2008 outbreak. Virol J 6:172. doi:10.1186/1743-422X-6-172. 19857273 PMC2774687

[22] Sumathy K, Ella KM. 2012. Genetic diversity of chikungunya virus, India 2006-2010: evolutionary dynamics and serotype analyses. J Med Virol 84:462–470. doi:10.1002/jmv.23187. 22246833

[23] Chen R, Puri V, Fedorova N, Lin D, Hari KL, Jain R, Rodas JD, Das SR, Shabman RS, Weaver SC. 2016. Comprehensive genome scale phylogenetic study provides new insights on the global expansion of chikungunya virus. J Virol 90:10600–10611. doi:10.1128/JVI.01166-16. 27654297 PMC5110187

[24] Tsetsarkin KA, Chen R, Yun R, Rossi SL, Plante KS, Guerbois M, Forrester N, Perng GC, Sreekumar E, Leal G, Huang J, Mukhopadhyay S, Weaver SC. 2014. Multi-peaked adaptive landscape for chikungunya virus evolution predicts continued fitness optimization in Aedes albopictus mosquitoes. Nat Commun 5:4084. doi:10.1038/ncomms5084. 24933611 PMC7091890

[25] Tsetsarkin KA, Weaver SC. 2011. Sequential adaptive mutations enhance efficient vector switching by chikungunya virus and its epidemic emergence. PLoS Pathog 7:e1002412. doi:10.1371/journal.ppat.1002412. 22174678 PMC3234230

[26] Voss JE, Vaney MC, Duquerroy S, Vonrhein C, Girard-Blanc C, Crublet E, Thompson A, Bricogne G, Rey FA. 2010. Glycoprotein organization of chikungunya virus particles revealed by X-ray crystallography. Nature 468:709–712. doi:10.1038/nature09555. 21124458

[27] Tsetsarkin KA, Chen R, Leal G, Forrester N, Higgs S, Huang J, Weaver SC. 2011. Chikungunya virus emergence is constrained in Asia by lineage-specific adaptive landscapes. Proc Natl Acad Sci USA 108:7872–7877. doi:10.1073/pnas.1018344108. 21518887 PMC3093459

[28] Tsetsarkin KA, McGee CE, Volk SM, Vanlandingham DL, Weaver SC, Higgs S. 2009. Epistatic roles of E2 glycoprotein mutations in adaption of chikungunya virus to Aedes albopictus and Ae. aegypti mosquitoes. PLoS One 4:e6835. doi:10.1371/journal.pone.0006835. 19718263 PMC2729410

[29] Modis Y, Ogata S, Clements D, Harrison SC. 2003. A ligand-binding pocket in the dengue virus envelope glycoprotein. Proc Natl Acad Sci USA 100:6986–6991. doi:10.1073/pnas.0832193100. 12759475 PMC165817

[30] Zhang Y, Zhang W, Ogata S, Clements D, Strauss JH, Baker TS, Kuhn RJ, Rossmann MG. 2004. Conformational changes of the flavivirus E glycoprotein. Structure 12:1607–1618. doi:10.1016/j.str.2004.06.019. 15341726 PMC4152830

[31] Teixeira MG, Andrade AMS, Costa MDCN, Castro J-NSM, Oliveira FLS, Goes CSB, Maia M, Santana EB, Nunes BTD, Vasconcelos PFC. 2015. East/Central/South African genotype chikungunya virus, Brazil, 2014. Emerg Infect Dis 21:906–907. doi:10.3201/eid2105.141727. 25898939 PMC4412231

[32] Kraemer MU, Sinka ME, Duda KA, Mylne AQ, Shearer FM, Barker CM, Moore CG, Carvalho RG, Coelho GE, Van Bortel W, Hendrickx G, Schaffner F, Elyazar IR, Teng HJ, Brady OJ, Messina JP, Pigott DM, Scott TW, Smith DL, Wint GR, Golding N, Hay SI. 2015. The global distribution of the arbovirus vectors Aedes aegypti and Ae. albopictus. Elife 4:e08347. doi:10.7554/eLife.08347. 26126267 PMC4493616

[33] Weaver SC, Charlier C, Vasilakis N, Lecuit M. 2018. Zika, chikungunya, and other emerging vector-borne viral diseases. Annu Rev Med 69:395–408. doi:10.1146/annurev-med-050715-105122. 28846489 PMC6343128

[34] Gay B, Bernard E, Solignat M, Chazal N, Devaux C, Briant L. 2012. pH-dependent entry of chikungunya virus into Aedes albopictus cells. Infect Genet Evol 12:1275–1281. doi:10.1016/j.meegid.2012.02.003. 22386853

[35] Lee RC, Hapuarachchi HC, Chen KC, Hussain KM, Chen H, Low SL, Ng LC, Lin R, Ng MM, Chu JJ. 2013. Mosquito cellular factors and functions in mediating the infectious entry of chikungunya virus. PLoS Negl Trop Dis 7:e2050. doi:10.1371/journal.pntd.0002050. 23409203 PMC3567007

[36] Hoornweg TE, van Duijl-Richter MKS, Ayala Nunez NV, Albulescu IC, van Hemert MJ, Smit JM. 2016. Dynamics of chikungunya virus cell entry unraveled by single-virus tracking in living cells. J Virol 90:4745–4756. doi:10.1128/JVI.03184-15. 26912616 PMC4836339

[37] van Duijl-Richter MK, Hoornweg TE, Rodenhuis-Zybert IA, Smit JM. 2015. Early events in chikungunya virus infection—from virus cell binding to membrane fusion. Viruses 7:3647–3674. doi:10.3390/v7072792. 26198242 PMC4517121

[38] Basore K, Kim AS, Nelson CA, Zhang R, Smith BK, Uranga C, Vang L, Cheng M, Gross ML, Smith J, Diamond MS, Fremont DH. 2019. Cryo-EM structure of chikungunya virus in complex with the Mxra8 receptor. Cell 177:1725–1737.e16. doi:10.1016/j.cell.2019.04.006. 31080061 PMC7227486

[39] Bonizzoni M, Gasperi G, Chen X, James AA. 2013. The invasive mosquito species Aedes albopictus: current knowledge and future perspectives. Trends Parasitol 29:460–468. doi:10.1016/j.pt.2013.07.003. 23916878 PMC3777778

[40] Tsetsarkin K, Higgs S, McGee CE, De Lamballerie X, Charrel RN, Vanlandingham DL. 2006. Infectious clones of chikungunya virus (La Réunion isolate) for vector competence studies. Vector Borne Zoonotic Dis 6:325–337. doi:10.1089/vbz.2006.6.325. 17187566

[41] Franz AW, Kantor AM, Passarelli AL, Clem RJ. 2015. Tissue barriers to arbovirus infection in mosquitoes. Viruses 7:3741–3767. doi:10.3390/v7072795. 26184281 PMC4517124

[42] Liu J, Liu Y, Shan C, Nunes BTD, Yun R, Haller SL, Rafael GH, Azar SR, Andersen CR, Plante K, Vasilakis N, Shi PY, Weaver SC. 2021. Role of mutational reversions and fitness restoration in Zika virus spread to the Americas. Nat Commun 12:595. doi:10.1038/s41467-020-20747-3. 33500409 PMC7838395

[43] Plante JA, Liu Y, Liu J, Xia H, Johnson BA, Lokugamage KG, Zhang X, Muruato AE, Zou J, Fontes-Garfias CR, Mirchandani D, Scharton D, Bilello JP, Ku Z, An Z, Kalveram B, Freiberg AN, Menachery VD, Xie X, Plante KS, Weaver SC, Shi PY. 2021. Spike mutation D614G alters SARS-CoV-2 fitness. Nature 592:116–121. doi:10.1038/s41586-020-2895-3. 33106671 PMC8158177

[44] Bergren NA, Haller S, Rossi SL, Seymour RL, Huang J, Miller AL, Bowen RA, Hartman DA, Brault AC, Weaver SC. 2020. “Submergence” of Western equine encephalitis virus: evidence of positive selection argues against genetic drift and fitness reductions. PLoS Pathog 16:e1008102. doi:10.1371/journal.ppat.1008102. 32027727 PMC7029877

[45] Carr IM, Robinson JI, Dimitriou R, Markham AF, Morgan AW, Bonthron DT. 2009. Inferring relative proportions of DNA variants from sequencing electropherograms. Bioinformatics 25:3244–3250. doi:10.1093/bioinformatics/btp583. 19819885

[46] Goddard TD, Huang CC, Meng EC, Pettersen EF, Couch GS, Morris JH, Ferrin TE. 2018. UCSF ChimeraX: meeting modern challenges in visualization and analysis. Protein Sci 27:14–25. doi:10.1002/pro.3235. 28710774 PMC5734306

[47] Pettersen EF, Goddard TD, Huang CC, Meng EC, Couch GS, Croll TI, Morris JH, Ferrin TE. 2021. UCSF ChimeraX: structure visualization for researchers, educators, and developers. Protein Sci 30:70–82. doi:10.1002/pro.3943. 32881101 PMC7737788

[48] Weaver SC, Forrester NL, Liu J, Vasilakis N. 2021. Population bottlenecks and founder effects: implications for mosquito-borne arboviral emergence. Nat Rev Microbiol 19:184–195. doi:10.1038/s41579-020-00482-8. PMC7798019 33432235

[49] Liu Y, Liu J, Plante KS, Plante JA, Xie X, Zhang X, Ku Z, An Z, Scharton D, Schindewolf C, Widen SG, Menachery VD, Shi P-Y, Weaver SC. The N501Y spike substitution enhances SARS-CoV-2 infection and transmission. Nature, in press. 10.1038/s41586-021-04245-0 PMC8900207 34818667

