# Supplementary material for: Lineage Divergence and Vector-Specific Adaptation Have Driven Chikungunya Virus onto Multiple Adaptive Landscapes
Source: mBio. 2021 Nov 9;12(6):e02738-21. doi: 10.1128/mBio.02738-21 (PMC8576524; doi:10.1128/mBio.02738-21)
Supplement: TABLE S1 [file mbio.02738-21-st001.pdf]

S1 Table: Distance in angstroms between amino acid residues that differ between Mal06 and SL07 glycoproteins

		E3					E2														E1					
		**18**	**33**	**44**	**60**	**62**	**2**	**5**	**118**	**157**	**164**	**194**	**198**	**205**	**207**	**210**	**233**	**248**	**252**	**255**	**72**	**98**	**196**	**203**	**211**	**225**
E3	18		>20	15	>20	>20	>20	15	>20	>20	>20	>20	>20	>20	>20	>20	>20	>20	>20	>20	>20	>20	>20	>20	>20	>20
	33			>20	15	>20	>20	>20	>20	>20	>20	>20	17	>20	>20	15	10	12	13	>20	>20	>20	>20	>20	>20	>20
	44				>20	>20	>20	>20	>20	>20	15	>20	>20	>20	>20	>20	>20	>20	>20	12	>20	>20	>20	>20	15	>20
	60					10	12	15	>20	>20	>20	>20	>20	>20	>20	>20	>20	>20	>20	>20	>20	>20	>20	>20	>20	>20
	62						15	15	>20	>20	>20	>20	>20	>20	>20	>20	>20	>20	>20	>20	>20	>20	>20	>20	>20	>20
E2	2							<6	>20	>20	>20	>20	>20	>20	>20	>20	>20	>20	>20	>20	>20	>20	>20	>20	>20	>20
	5								>20	>20	>20	>20	>20	>20	>20	>20	>20	>20	>20	>20	>20	>20	>20	>20	>20	>20
	118									>20	>20	>20	>20	>20	>20	>20	>20	>20	>20	>20	>20	>20	>20	>20	>20	>20
	157										11	>20	>20	>20	>20	>20	>20	>20	>20	15	>20	>20	>20	>20	>20	>20
	164											>20	>20	>20	>20	>20	>20	>20	>20	10	>20	>20	>20	>20	15	>20
	194												15	>20	>20	12	<10	>20	>20	>20	>20	>20	>20	>20	>20	>20
	198													15	>20	18	>20	>20	>20	>20	>20	>20	>20	>20	>20	>20
	205														>20	12	>20	>20	>20	>20	>20	18 flex	>20	>20	>20	>20
	207															14	>20	>20	>20	>20	>20	15	>20	>20	>20	>20
	210																>20	16	>20	>20	>20	>20	>20	>20	>20	>20
	233																	10	15	>20	>20	>20	>20	>20	>20	>20
	248																		10	15	>20	>20	>20	>20	>20	>20
	252																			12	17	>20	>20	>20	16	>20
	255																				>20	>20	>20	>20	>20	>20
E1	72																					>20	>20	15	13	>20
	98																						>20	15	>20	>20
	196																							10	>20	>20
	203																								12	>20
	211																									>20
	225																									

The 35 amino acid residues that differ between IOL (SL07) and Asian (Mal06) CHIKV were analyzed *in silico* to measure their inter-residue distance using structure 3N40 in PyMol. Residues which differed between SL07 and Mal06 but were missing or incompletely resolved in 3N40 were excluded from analysis. Distances of <20 angstroms are highlighted in green.
