# Supplementary material for: Lineage Divergence and Vector-Specific Adaptation Have Driven Chikungunya Virus onto Multiple Adaptive Landscapes
Source: mBio. 2021 Nov 9;12(6):e02738-21. doi: 10.1128/mBio.02738-21 (PMC8576524; doi:10.1128/mBio.02738-21)
Supplement: TABLE S2 [file mbio.02738-21-st002.pdf]

S2 Table. Envelope protein amino acids that differ between IOL (SL07) and Asian (Mal06) CHIKV strains and potentially interact with the IOL adaptive E2 mutations.

Protein	Residue	Amino Acid		Distance from E2 Residue (Å)			
		SL07	Mal06	198	210	233	252
E3	33	E	K	17	15	10	13
E2	194	G	S	15	12	<10	>20
	205	G	D	15	12	>20	>20
	207	N	S	>20	14	>20	>20
	248	L	S	>20	16	10	10
	255	I	V	>20	>20	>20	12
E1	72	N	S	>20	>20	>20	17
	211	K	E	>20	>20	>20	16

Summary of residues which both (a) differ between IOL (SL07) and Asian (Mal06) CHIKV, and (b) are located within 20 angstrom of at least one of the E2 adaptive residues 198, 210, 233, and/or 252. Distances of <20 angstrom are highlighted in green.
