# Supplementary material for: Lineage Divergence and Vector-Specific Adaptation Have Driven Chikungunya Virus onto Multiple Adaptive Landscapes
Source: mBio. 2021 Nov 9;12(6):e02738-21. doi: 10.1128/mBio.02738-21 (PMC8576524; doi:10.1128/mBio.02738-21)

Figure S1: Passage of competing viruses in Vero Cells. All competing groups were mixed in 3 different ratios and passaged in Vero cells in 96 well plates in duplicate for 48 hours. RNA from the initial mix and passaged material purified, amplified via RT-PCR, and restriction digested as described in the materials and methods. Gel lanes are labeled as I for the input and 1 and 2 for the duplicate passages. Proportions of DNA as estimated by GelQuant.NET software are shown in red next to the relevant bands.

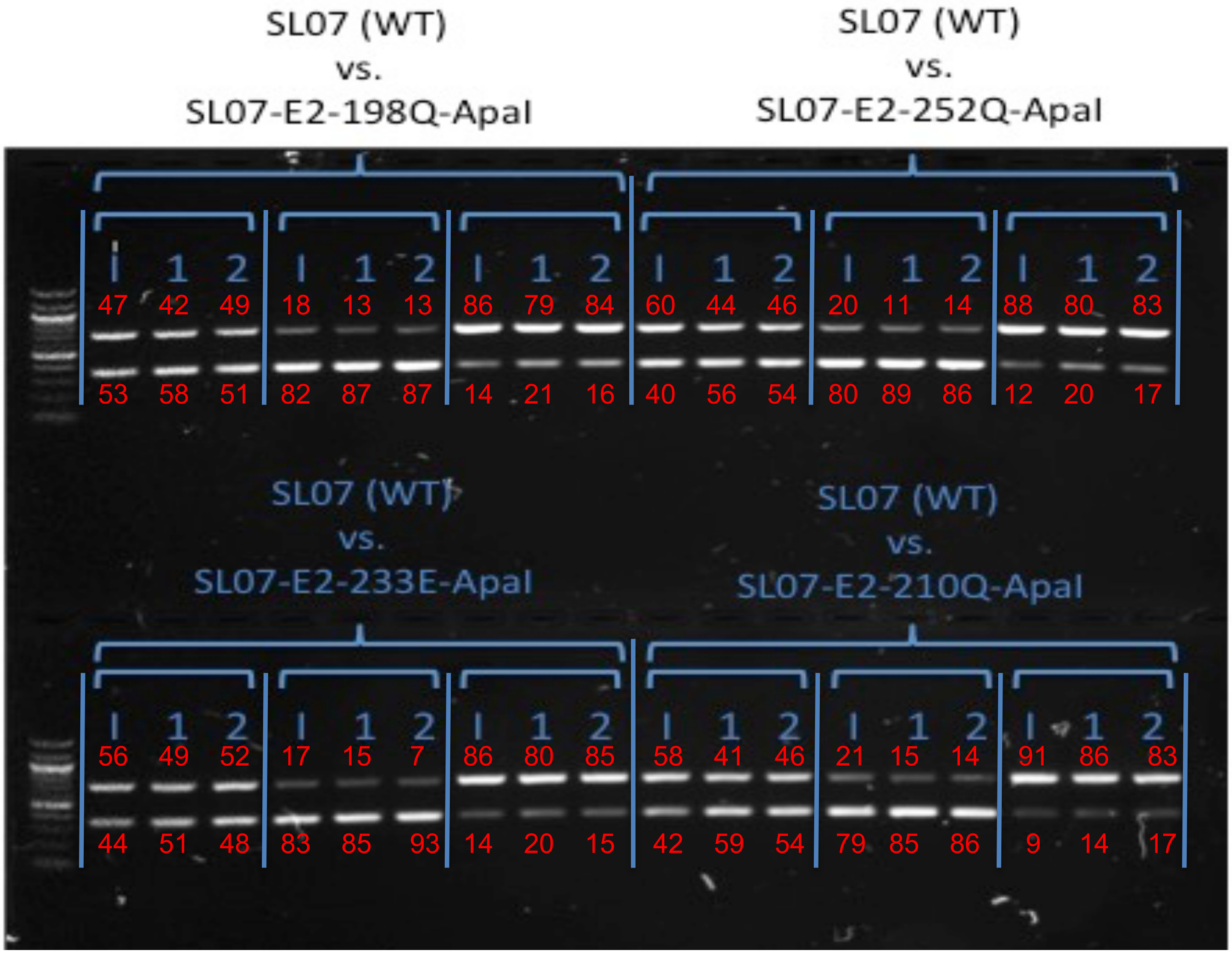

SL07-E2-210Q
vs.
SL07 (WT+ApaI)

SL07-E2-198Q
vs.
SL07 (WT+ApaI)

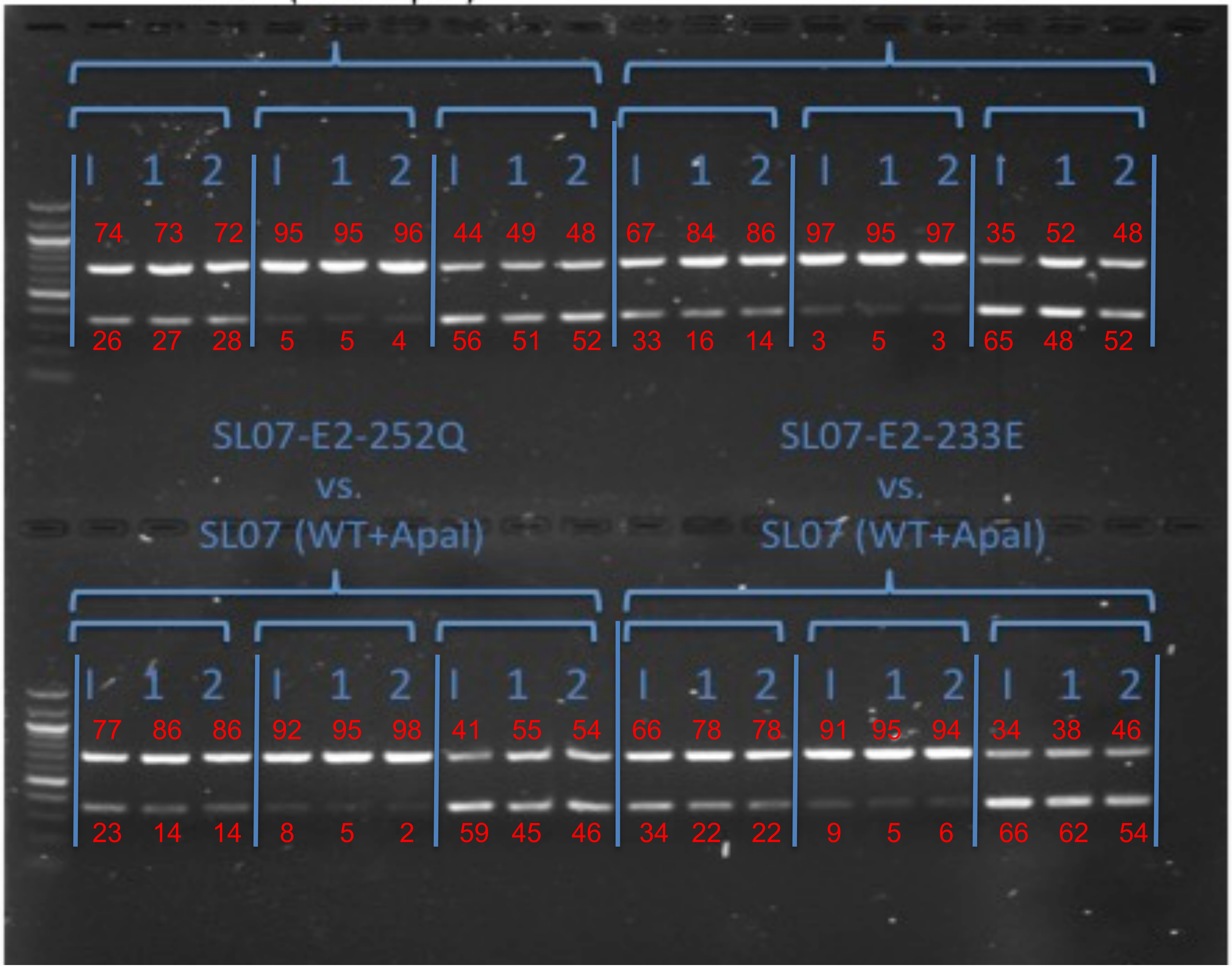

Figure S1: Passage of competing viruses in Vero Cells (Continued).

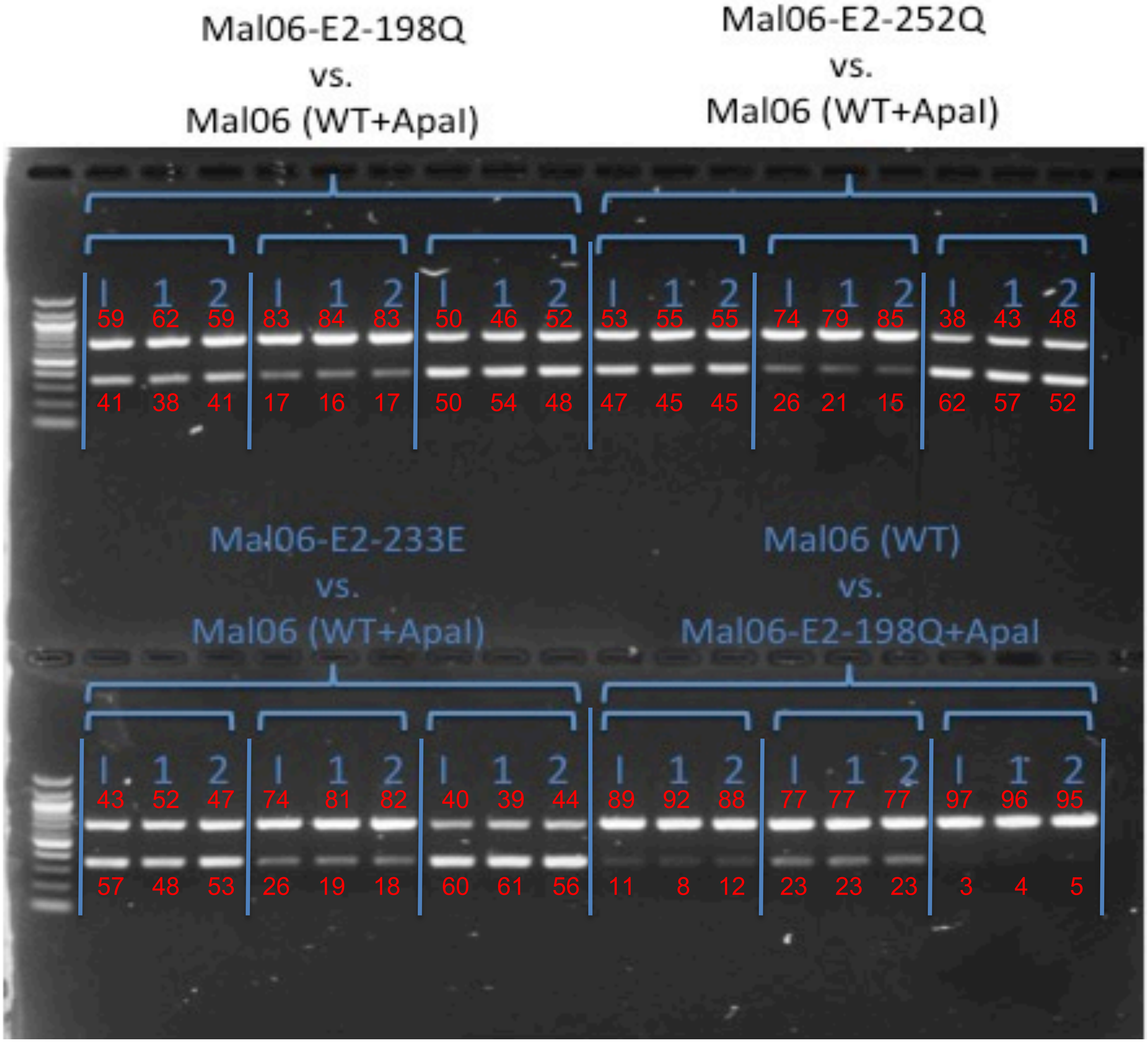

Figure S1: Passage of competing viruses in Vero Cells (Continued).

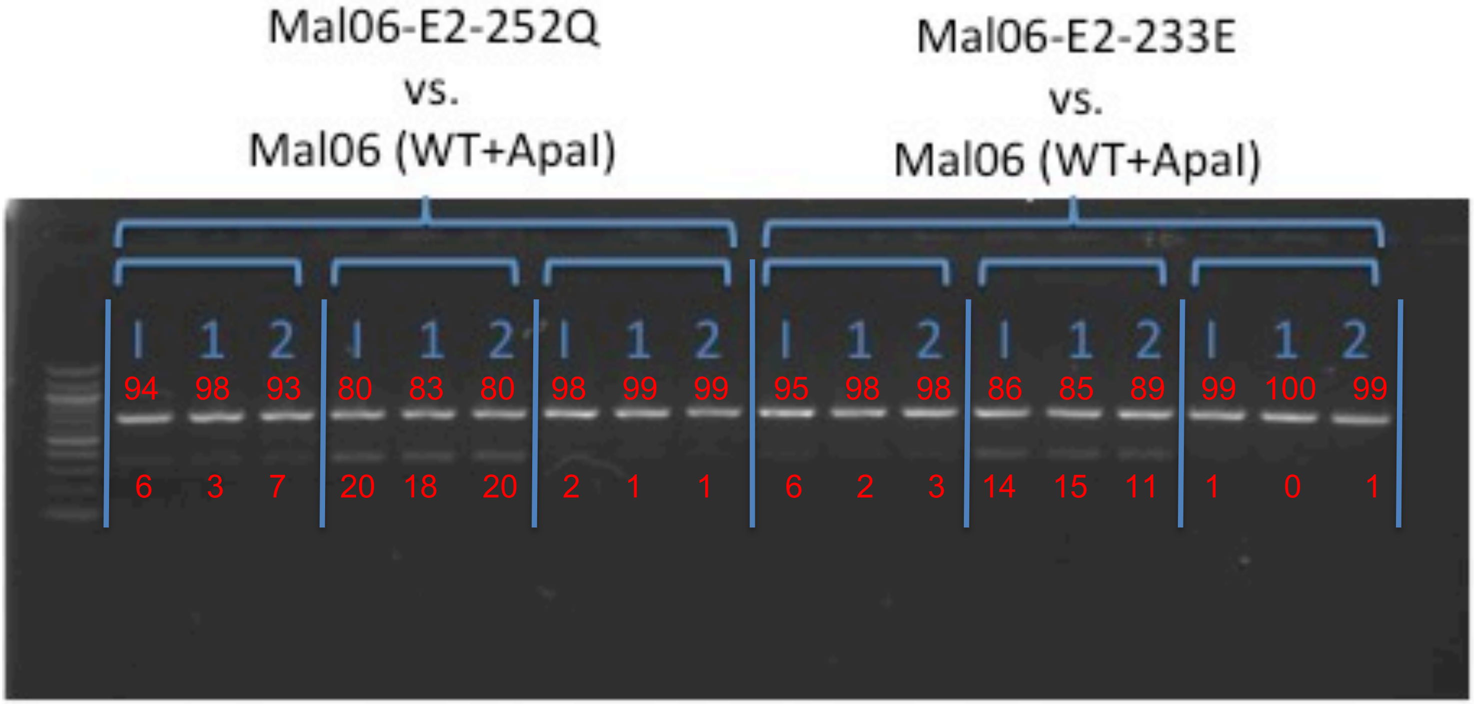

Supplement: FIG S1 [file mbio.02738-21-sf001.pdf]
