# Supplementary material for: Lineage Divergence and Vector-Specific Adaptation Have Driven Chikungunya Virus onto Multiple Adaptive Landscapes
Source: mBio. 2021 Nov 9;12(6):e02738-21. doi: 10.1128/mBio.02738-21 (PMC8576524; doi:10.1128/mBio.02738-21)

Figure S2: Gel images of competition test results. The raw data corresponding to Figures 1 and 2 are shown here. For each gel picture, the digestion result of the initial mix of viruses is shown in the first lane (I). The following lanes show the results in each mosquito sample (M#). Proportions of DNA as estimated by GelQuant.NET software are shown in red next to the relevant bands.

SL07 (WT) vs SL07-E2-198Q-ApaI

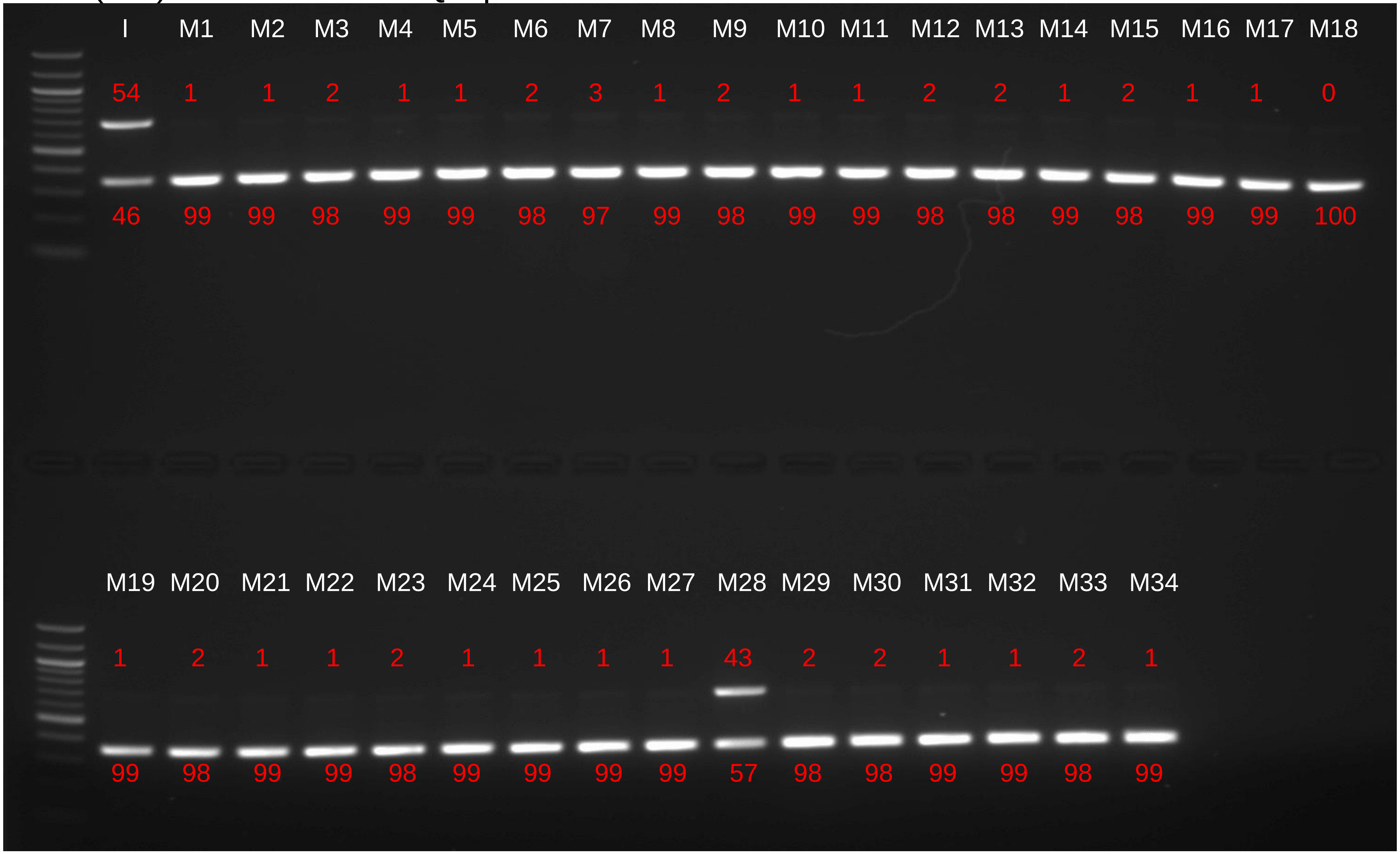

Figure S2: Gel images of competition test results (continued).

SL07 (WT) vs SL07-E2-210Q-ApaI

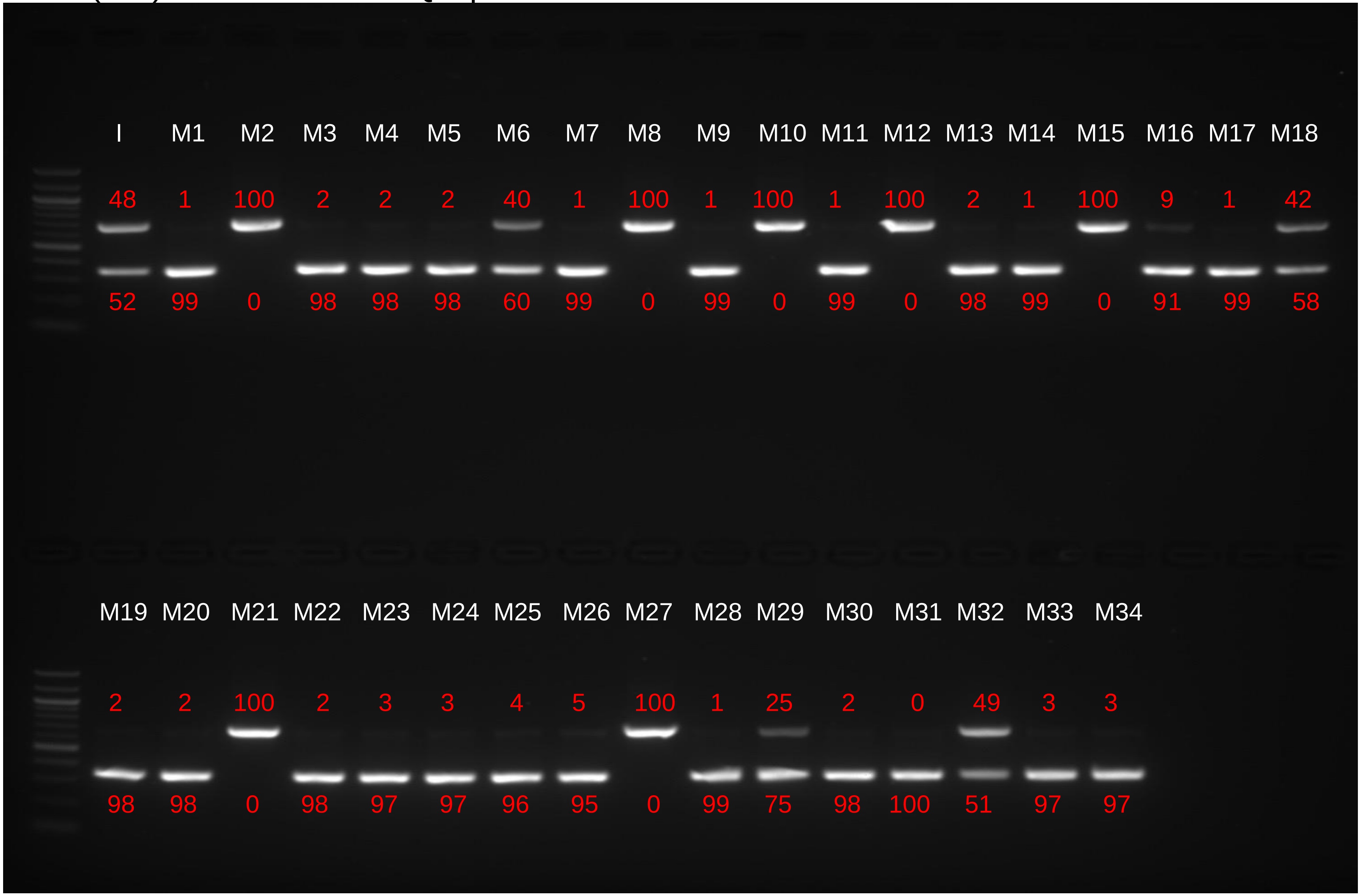

Figure S2: Gel images of competition test results (continued).

SL07 (WT) vs SL07-E2-233E-ApaI

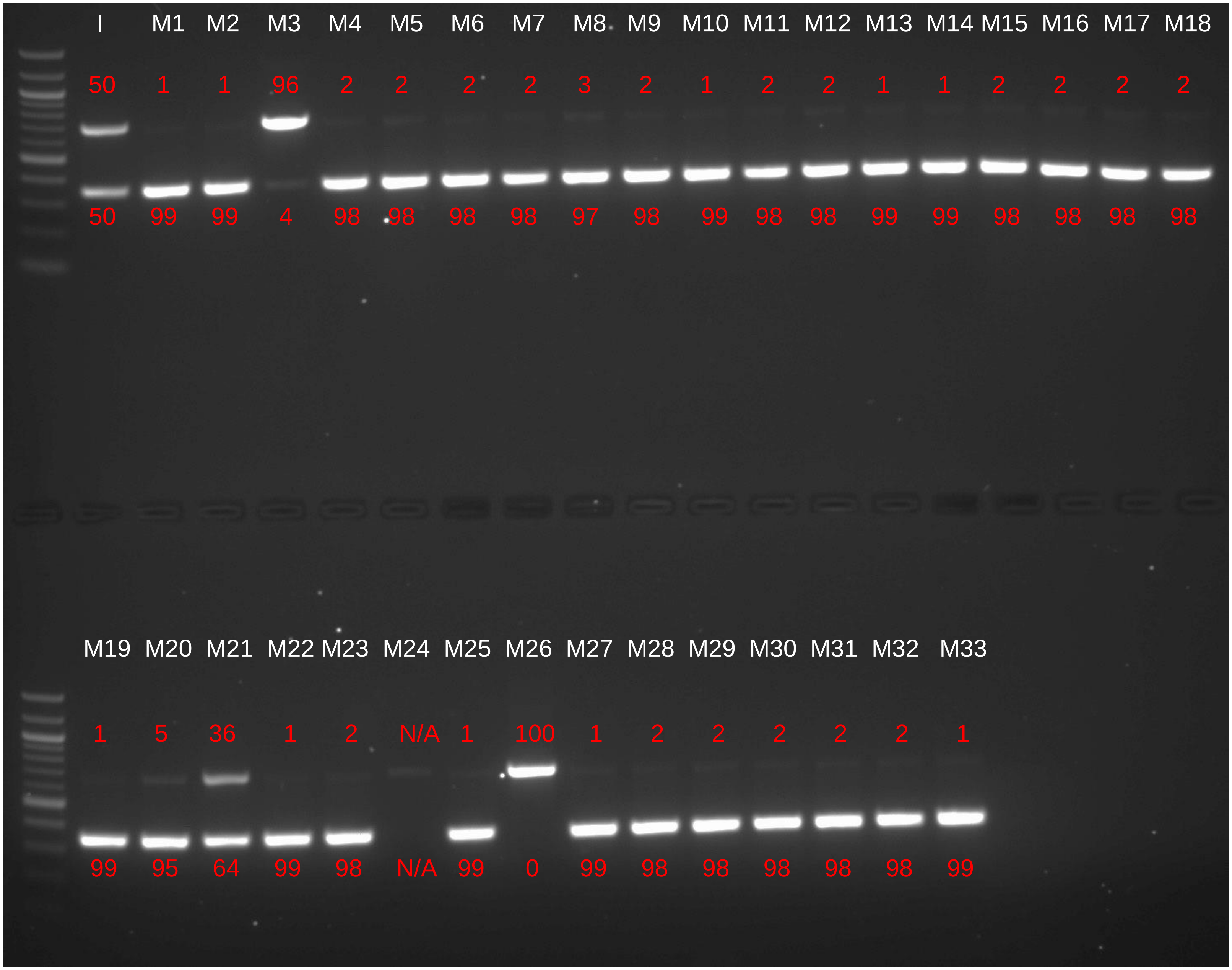

Figure S2: Gel images of competition test results (continued).

SL07 (WT) vs SL07-E2-252Q-ApaI

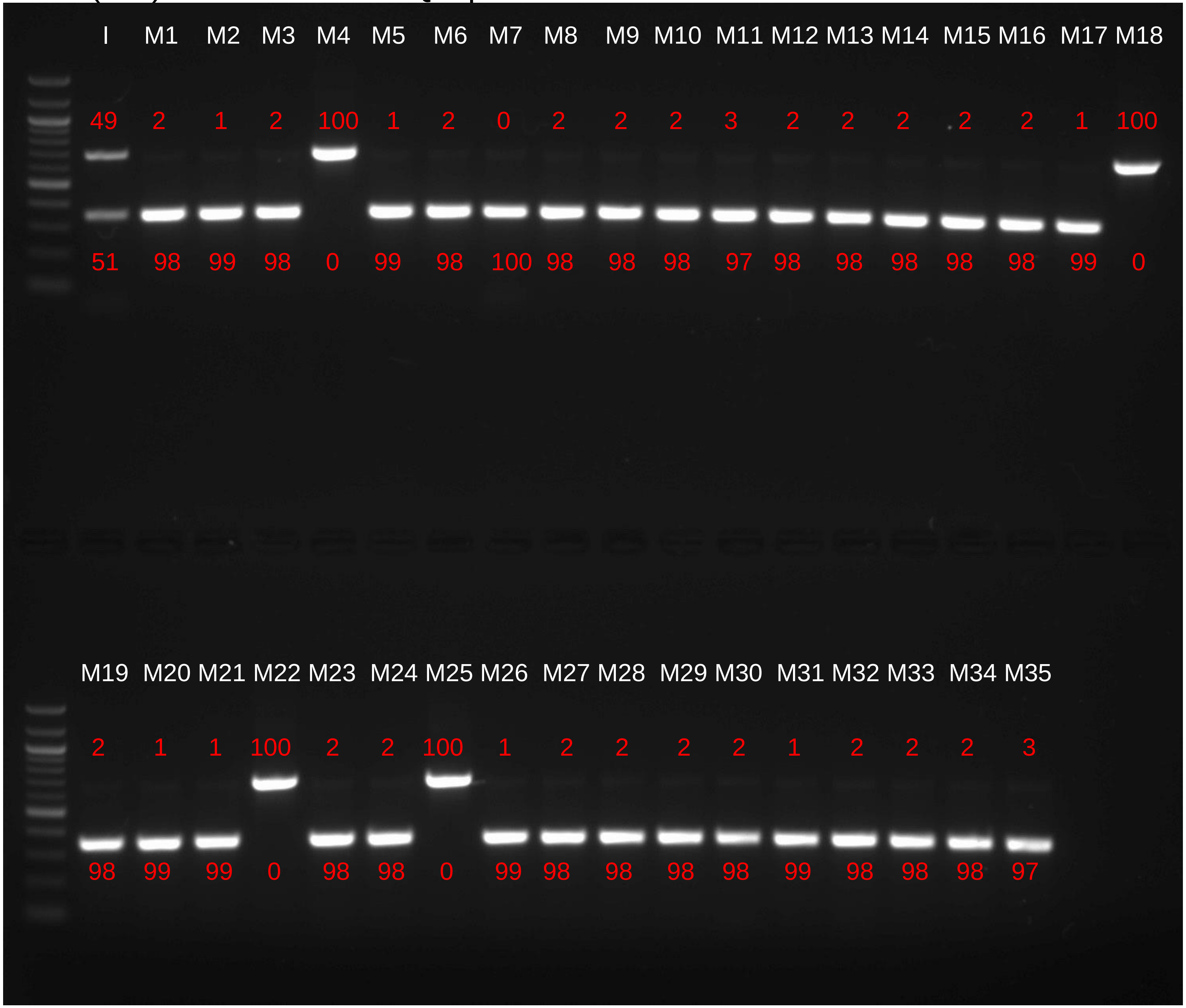

Figure S2: Gel images of competition test results (continued).

SL07-E2-198Q vs SL07 (WT+ApaI)

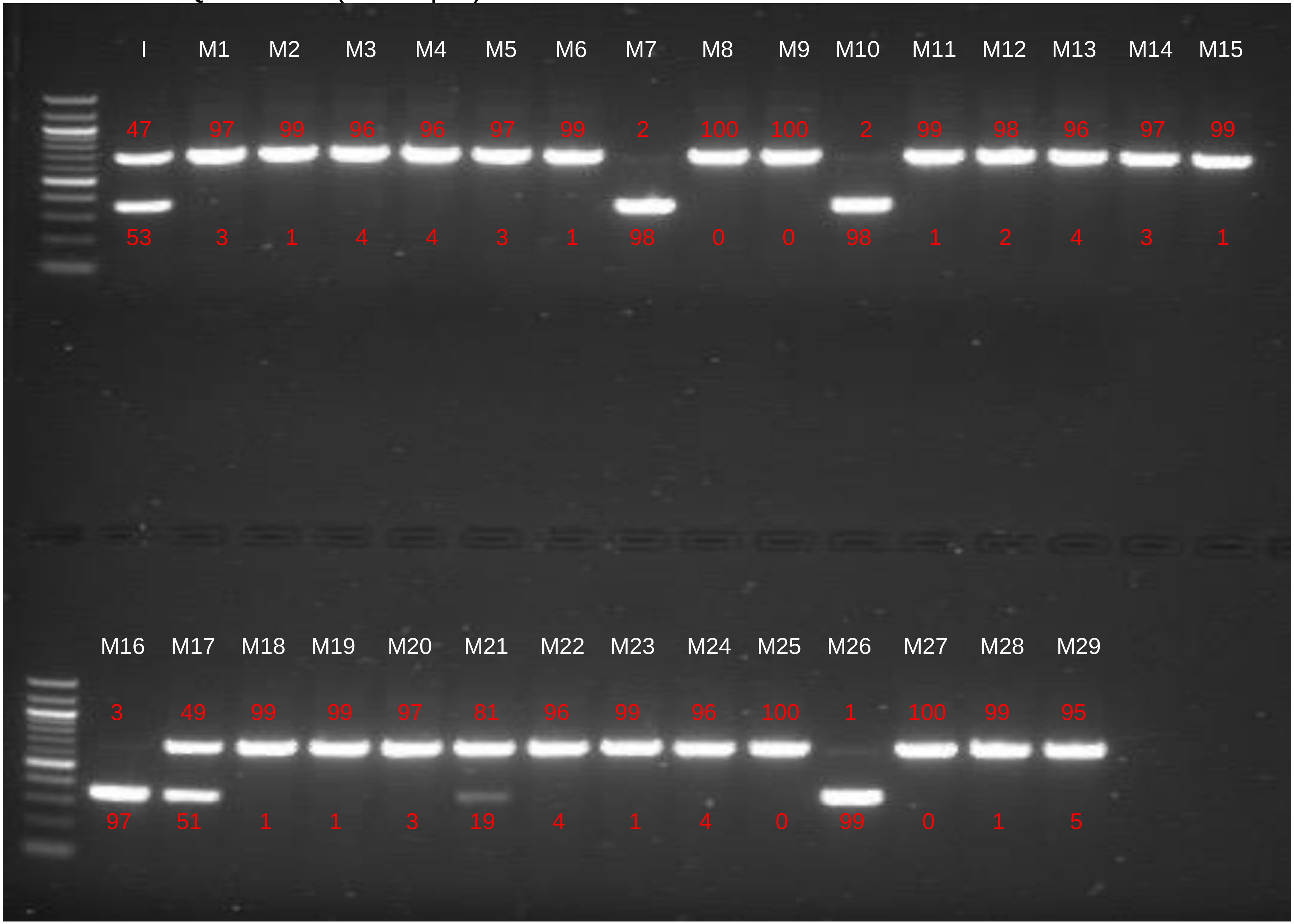

Figure S2: Gel images of competition test results (continued).

SL07-E2-210Q vs SL07 (WT+ApaI)

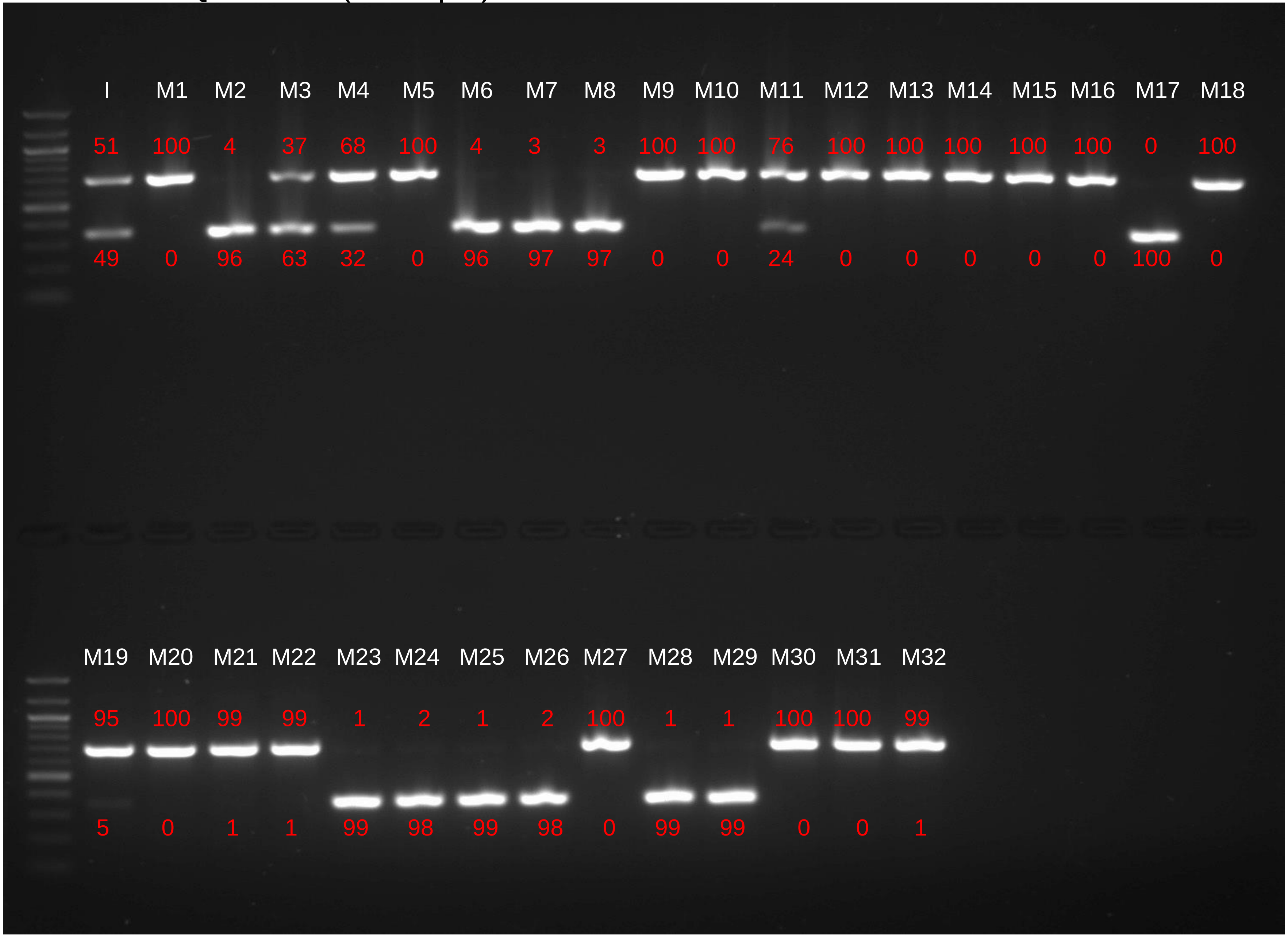

Figure S2: Gel images of competition test results (continued).

SL07-E2-233E vs SL07 (WT+ApaI)

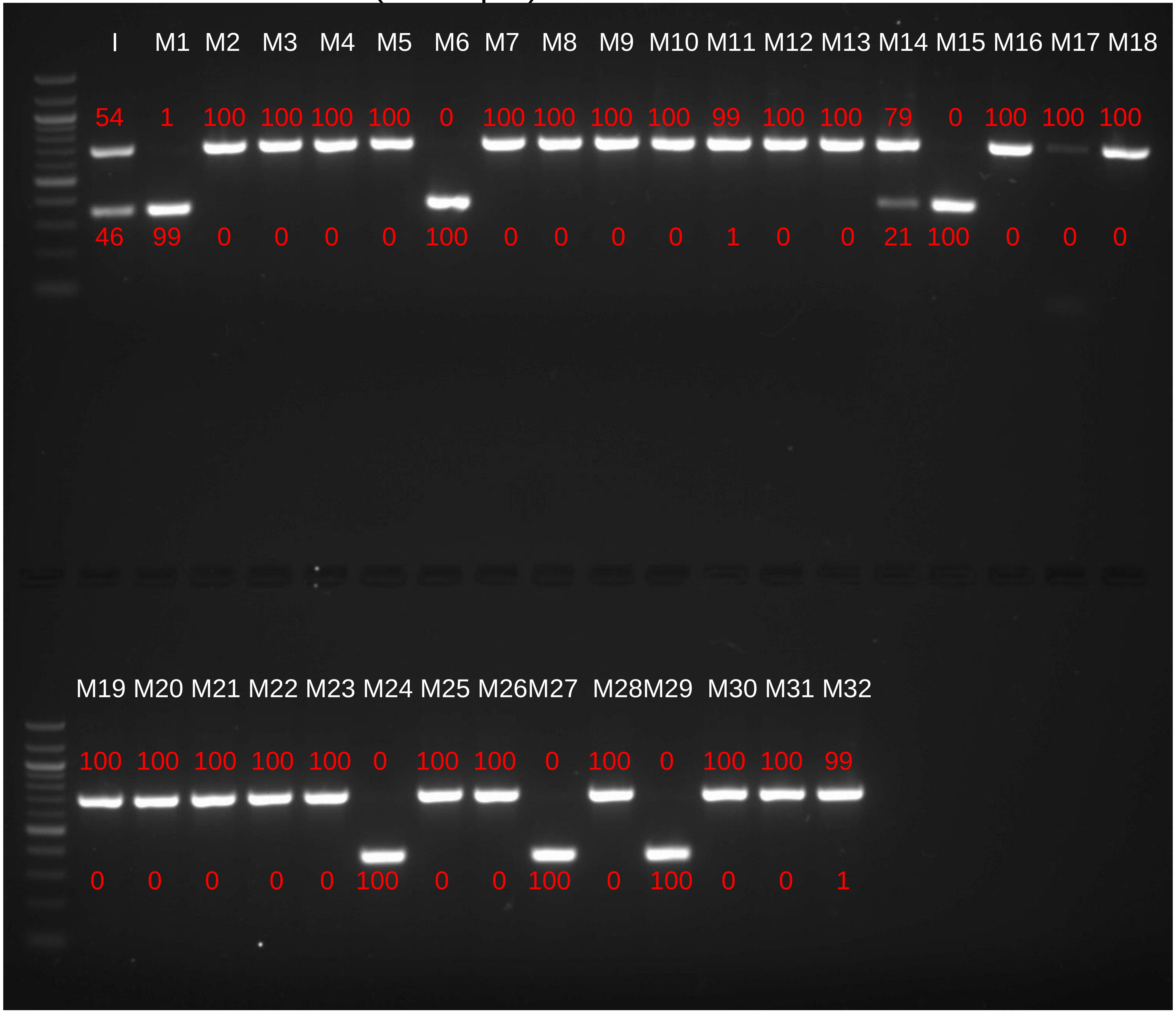

Figure S2: Gel images of competition test results (continued).

SL07-E2-252Q vs SL07 (WT+ApaI)

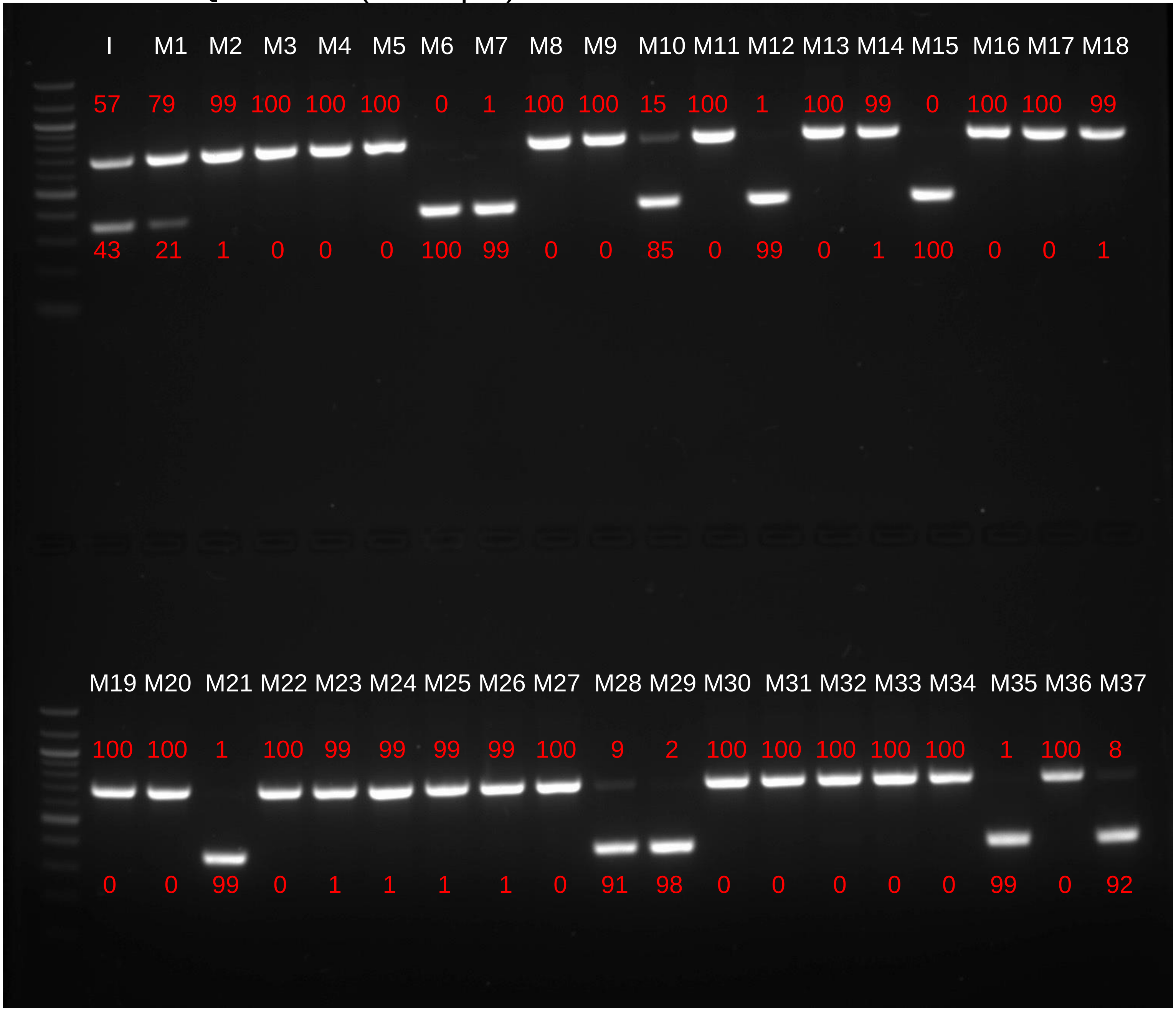

Figure S2: Gel images of competition test results (continued).

Mal06 (WT) vs Mal06-E2-198Q+ApaI

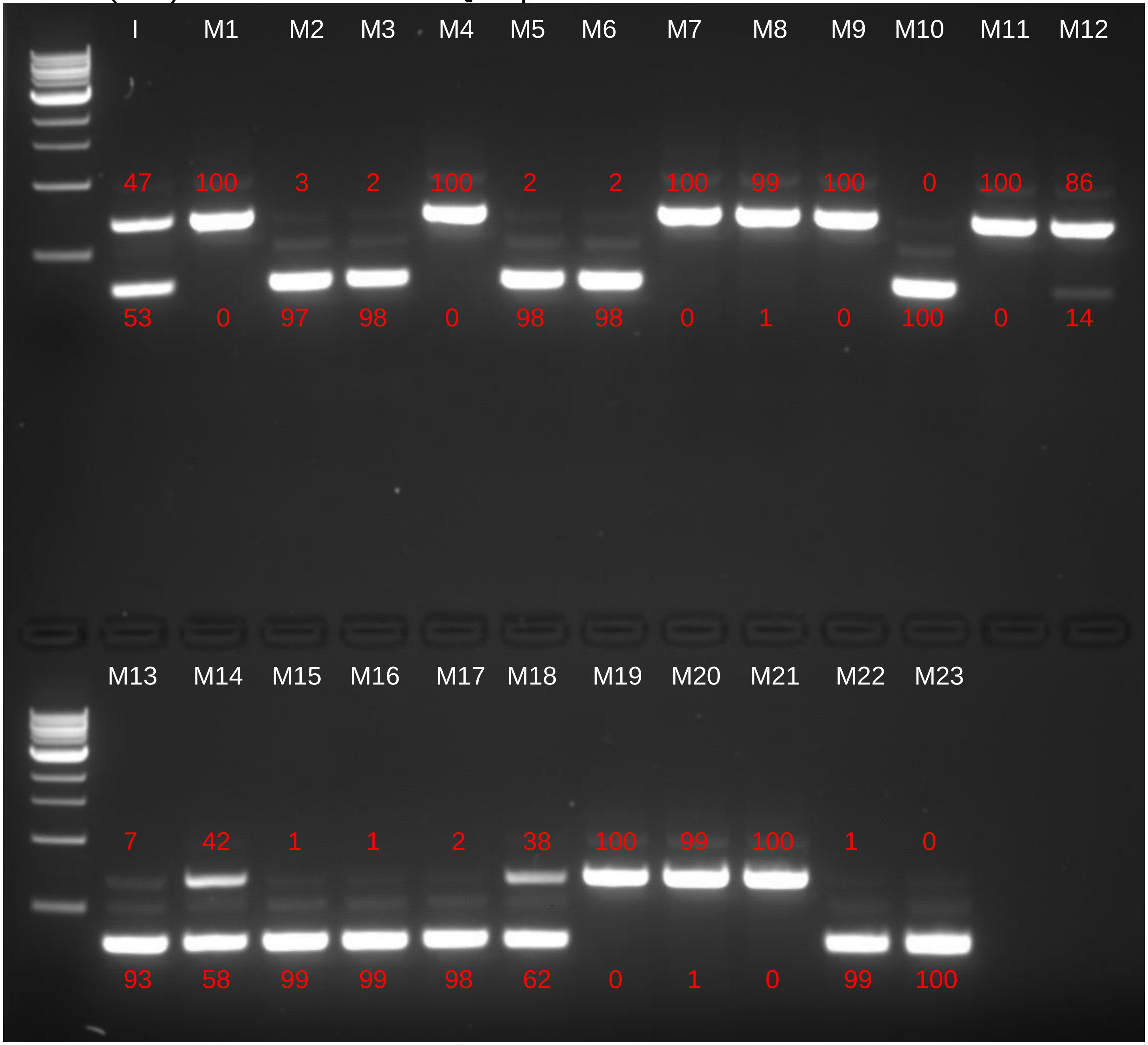

Figure S2: Gel images of competition test results (continued).

Mal06 (WT) vs Mal06-E2-233E+ApaI

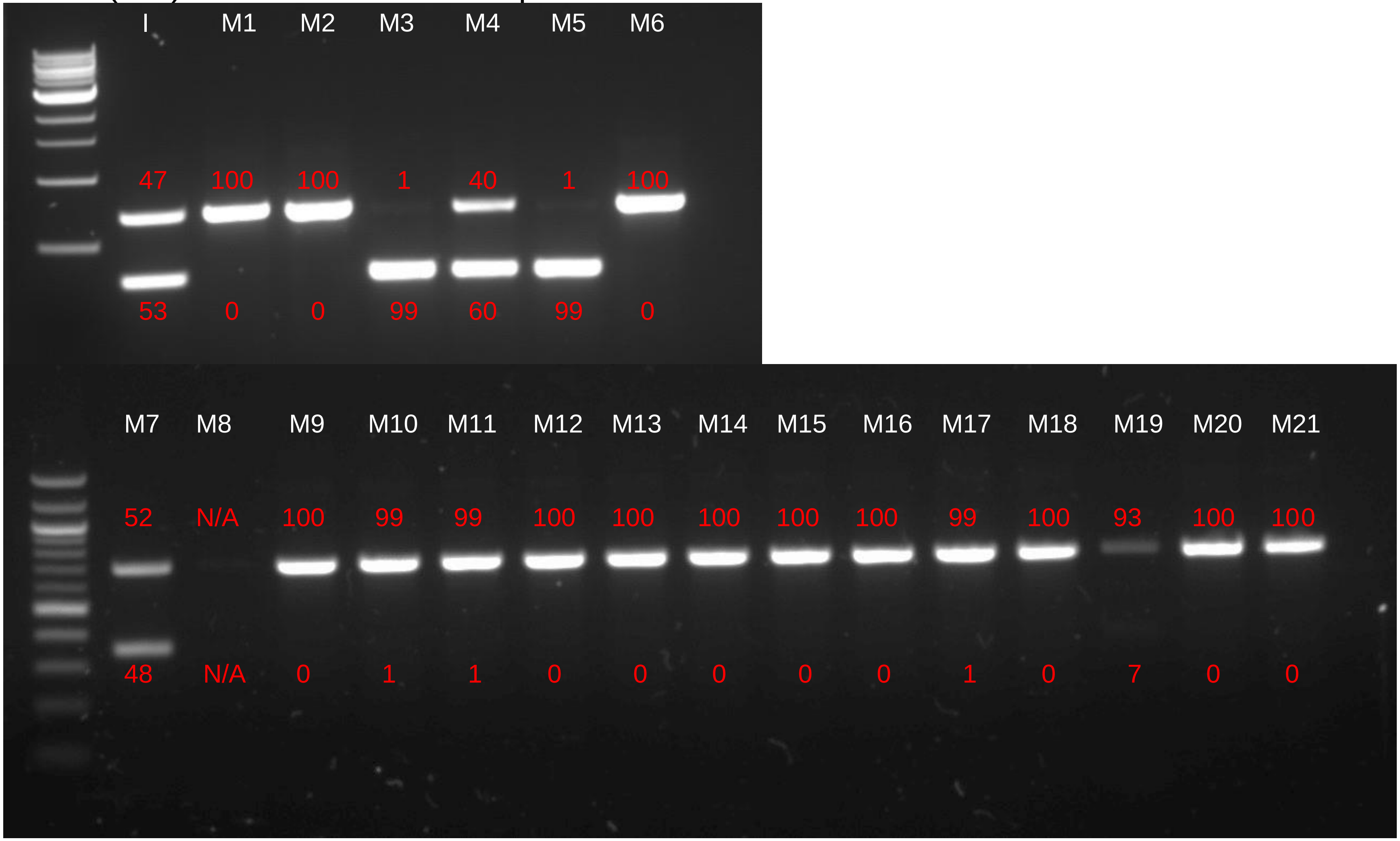

Figure S2: Gel images of competition test results (continued).

Mal06 (WT) vs Mal06-E2-252Q+ApaI

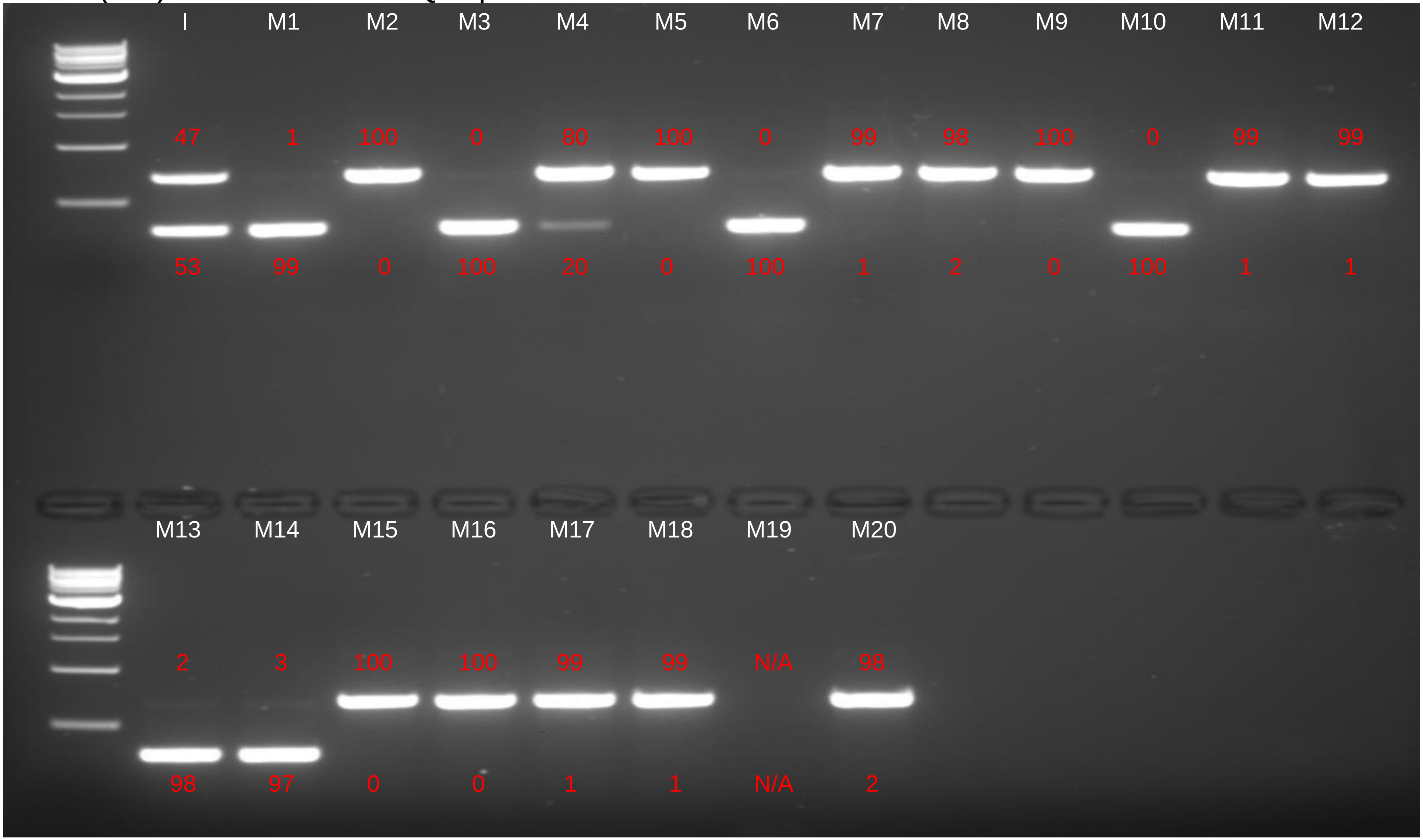

Figure S2: Gel images of competition test results (continued).

Mal06-E2-198Q vs Mal06 (WT+ApaI)

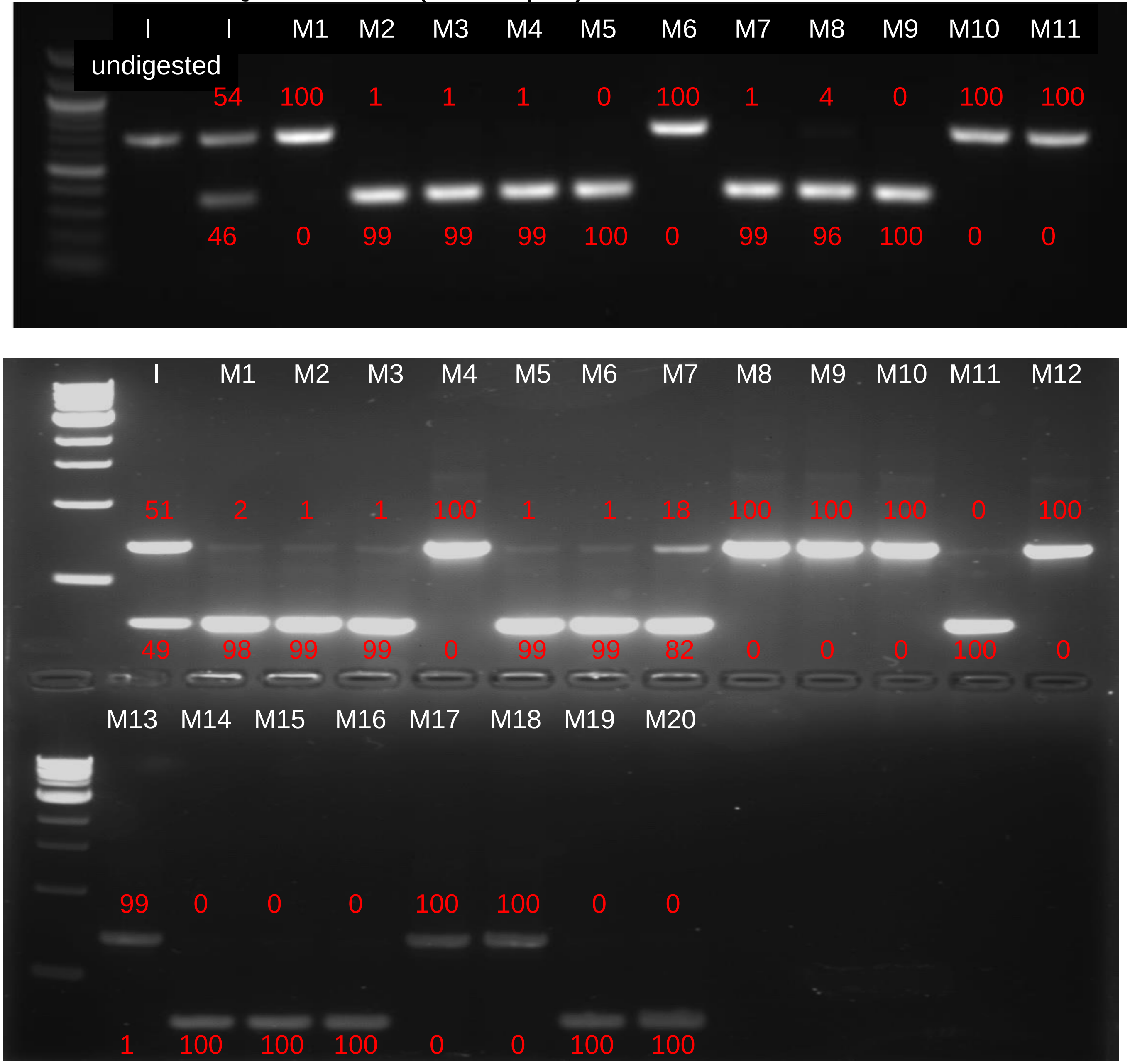

Figure S2: Gel images of competition test results (continued).

Mal06-E2-233E vs Mal06 (WT+ApaI)

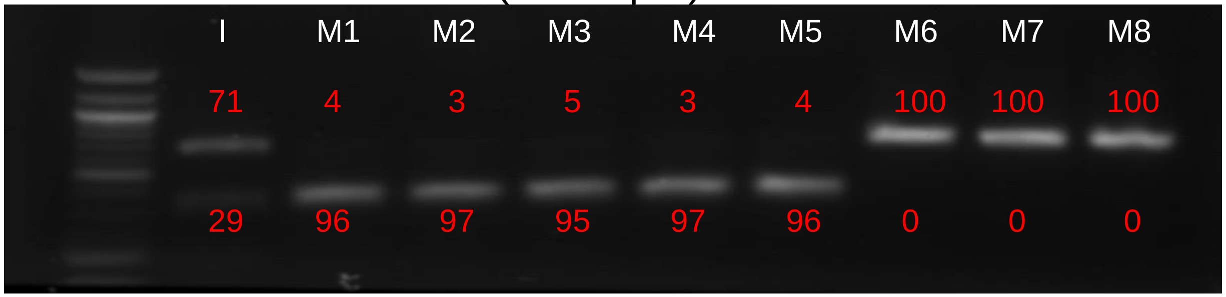

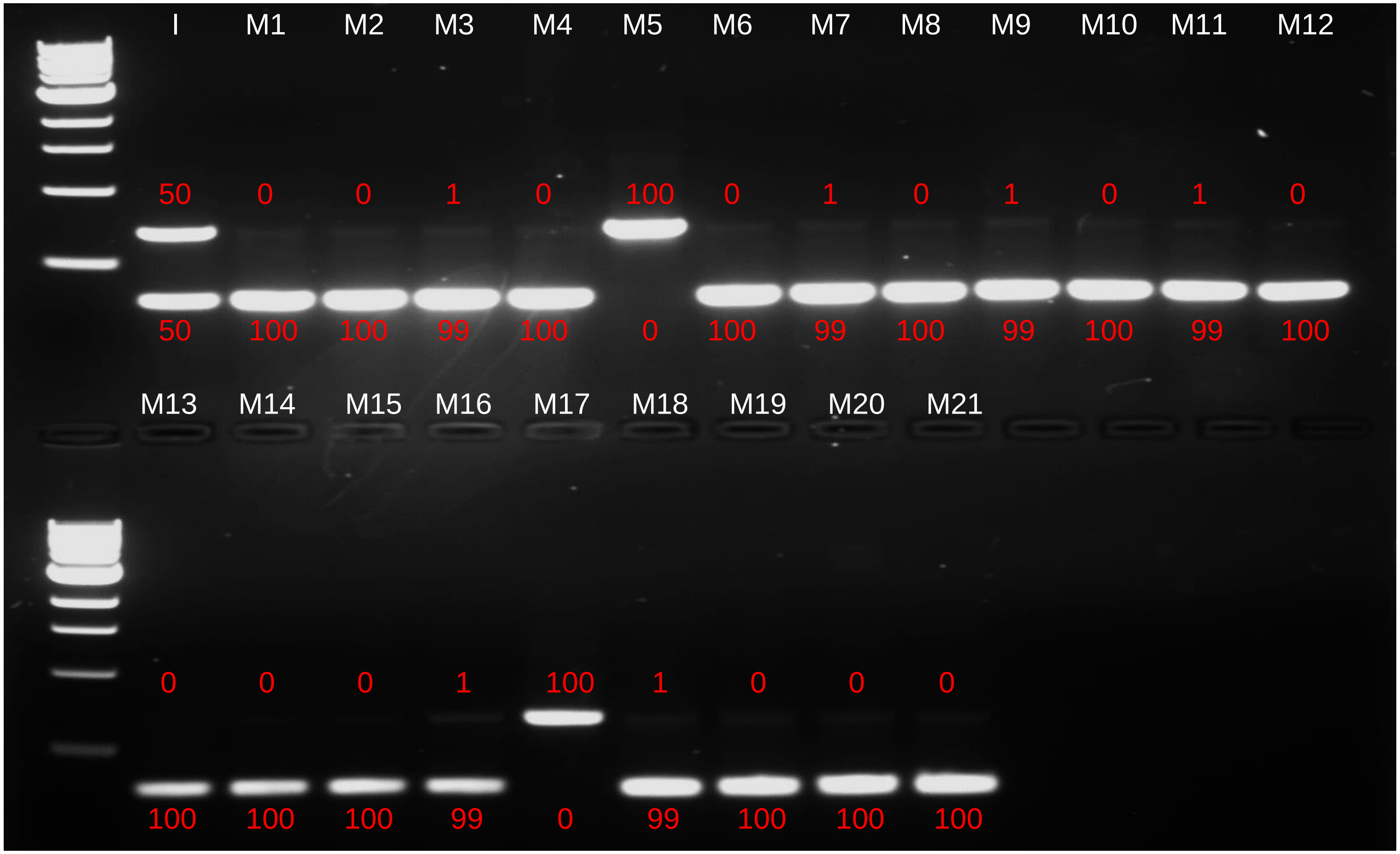

Figure S2: Gel images of competition test results (continued).

Mal06-E2-252Q vs Mal06 (WT+ApaI)

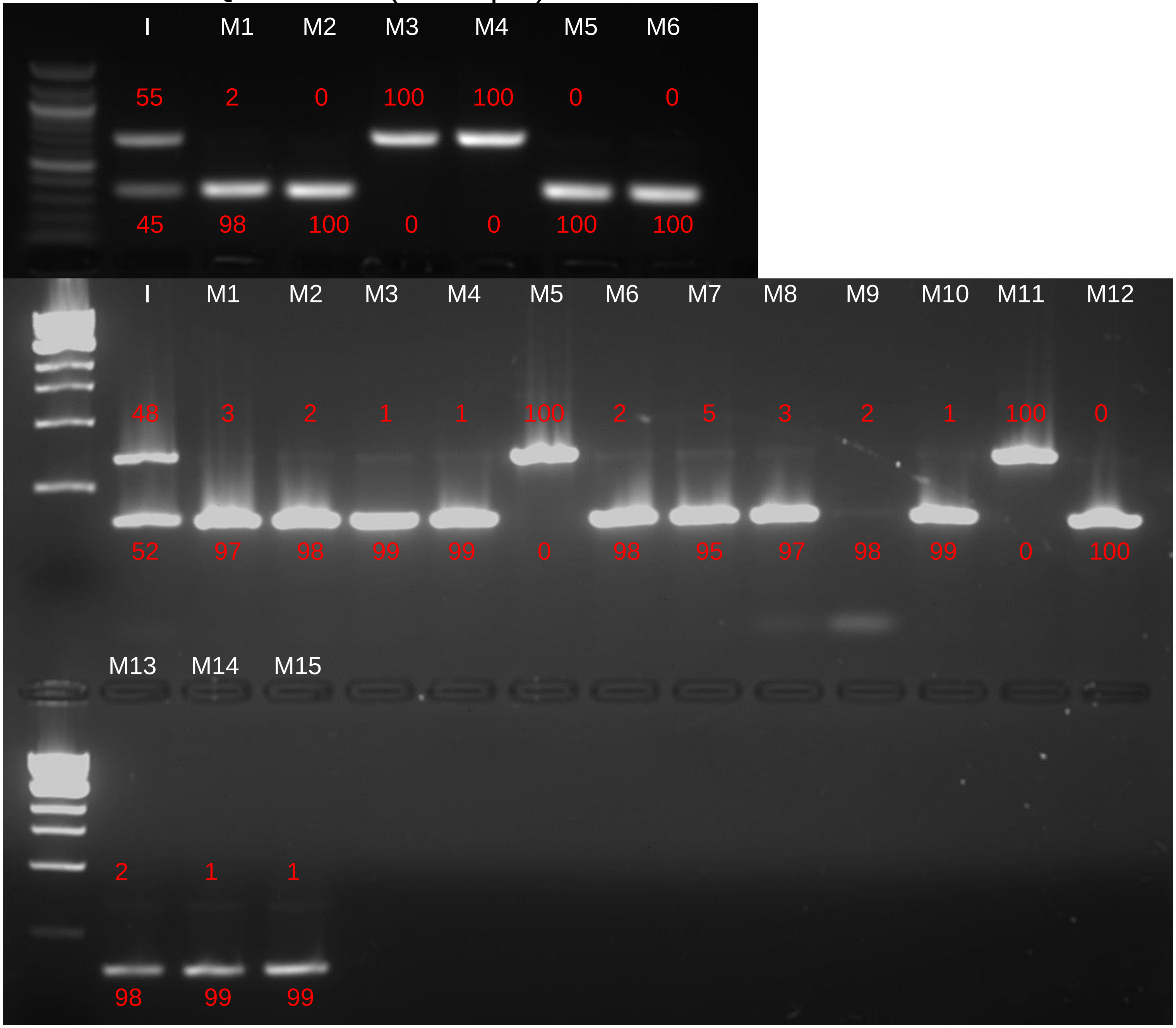

Supplement: FIG S2 [file mbio.02738-21-sf002.pdf]
