# Supplementary material for: Lineage Divergence and Vector-Specific Adaptation Have Driven Chikungunya Virus onto Multiple Adaptive Landscapes
Source: mBio. 2021 Nov 9;12(6):e02738-21. doi: 10.1128/mBio.02738-21 (PMC8576524; doi:10.1128/mBio.02738-21)
Supplement: FIG S3 [file mbio.02738-21-sf003.pdf]

Figure S3: Validation of Restriction Digest-Based Genome Ratio Assessments

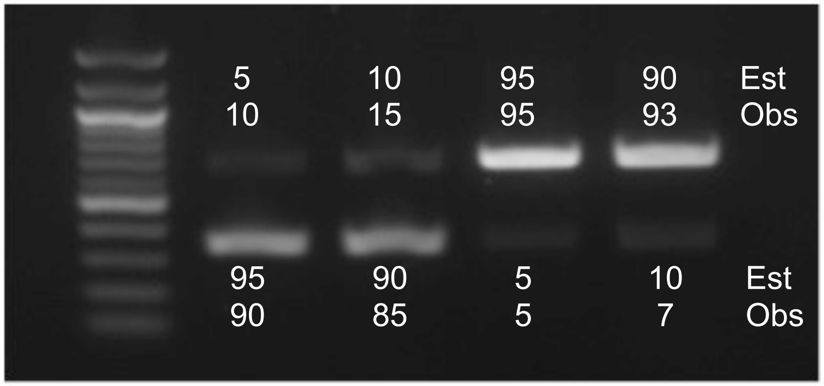

CHIKV SL07 RNA, either wild-type containing no restriction enzyme marker or with the E2-R198Q mutation and the ApaI marker, was extracted from viral stocks and combined at four different ratios. The RNA mixtures were subjected to RT-PCR and restriction digest analysis as described in the materials and methods. Estimated ratios (on the basis of RNA input as determined by empirically determining the ratio required for a 50:50 observed banding pattern and then scaling the ratios to 5:95, 10:90, 95:5, and 90:10) and observed ratios (on the basis of GelQuant.net software) are noted above and below the relevant bands. An unpaired, two-tailed Student’s t-test of the mutant:wild type band ratios found no statistically significant difference between the estimated values and the observed values (p=0.530101, ns).
