# Supplementary material for: Lineage Divergence and Vector-Specific Adaptation Have Driven Chikungunya Virus onto Multiple Adaptive Landscapes
Source: mBio. 2021 Nov 9;12(6):e02738-21. doi: 10.1128/mBio.02738-21 (PMC8576524; doi:10.1128/mBio.02738-21)
Supplement: FIG S4 [file mbio.02738-21-sf004.pdf]

Figure S4: Validation of Sanger-Based Genome Ratio Assessments

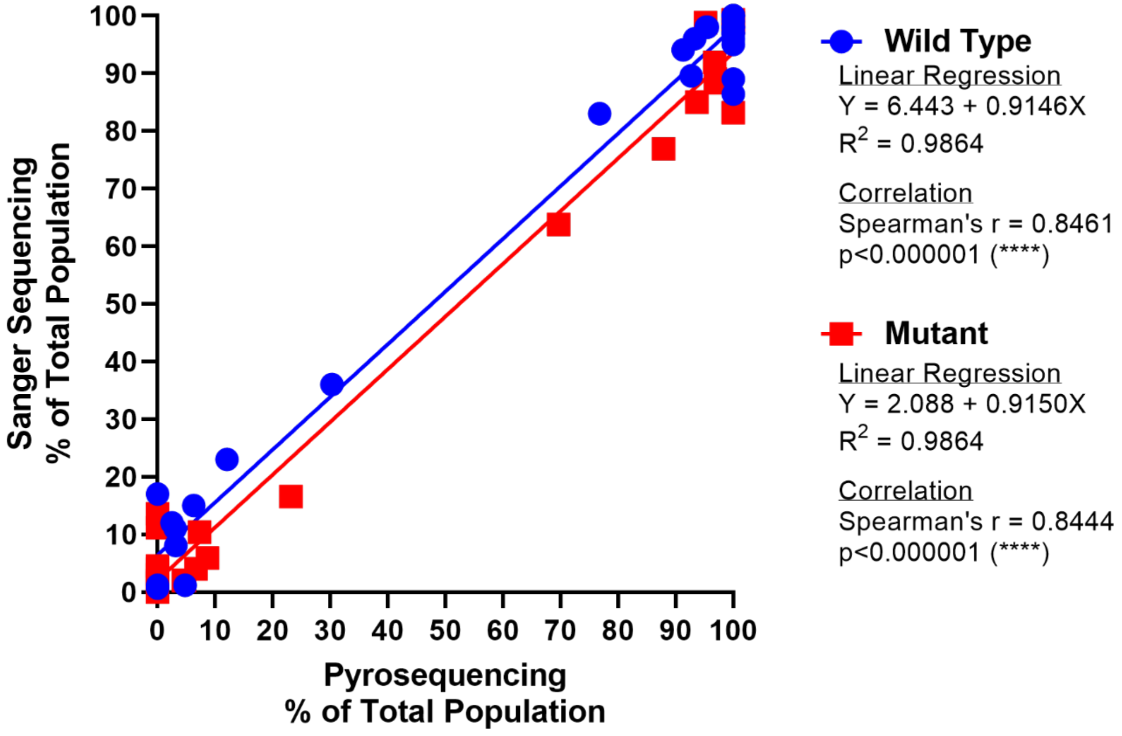

Aedes albopictus (Thailand) mosquitoes were fed chikungunya virus (CHIKV) mixtures of strain SL07 wild type and either E2-R198Q, E2-L210Q, E2-K333E, or E2-K252Q at approximately equal ratios (mean = 1:1.31, median = 1:1.17) and a combined concentration of $1x10^7$ PFU/ml. After ten days, bodies and heads were harvested. Bodies were screened for the presence of CHIKV by CPE assay. Eight heads per cohort were selected, each with a CHIKV-positive body. RNA was extracted from the heads, and subjected to both Sanger sequencing (as described in the methods section) and pyrosequencing. The correlation between each method's detected percentage of wild type and mutant genomes were assessed by linear regression and by Spearman's correlation.
